# Supplementary material for: A conserved ubiquitin- and ESCRT-dependent pathway internalizes human lysosomal membrane proteins for degradation
Source: PLoS Biol. 2021 Jul 23;19(7):e3001361. doi: 10.1371/journal.pbio.3001361 (PMC8337054; doi:10.1371/journal.pbio.3001361)
Supplement: S2 Data — An example of the gating applied to FCS files in Fig 2D. FCS, Flow Cytometry Standard. (PDF) [file pbio.3001361.s009.pdf]

Example of Gates applied to the FCS files (Fig 2D)
HEK293, GFP-RNF152

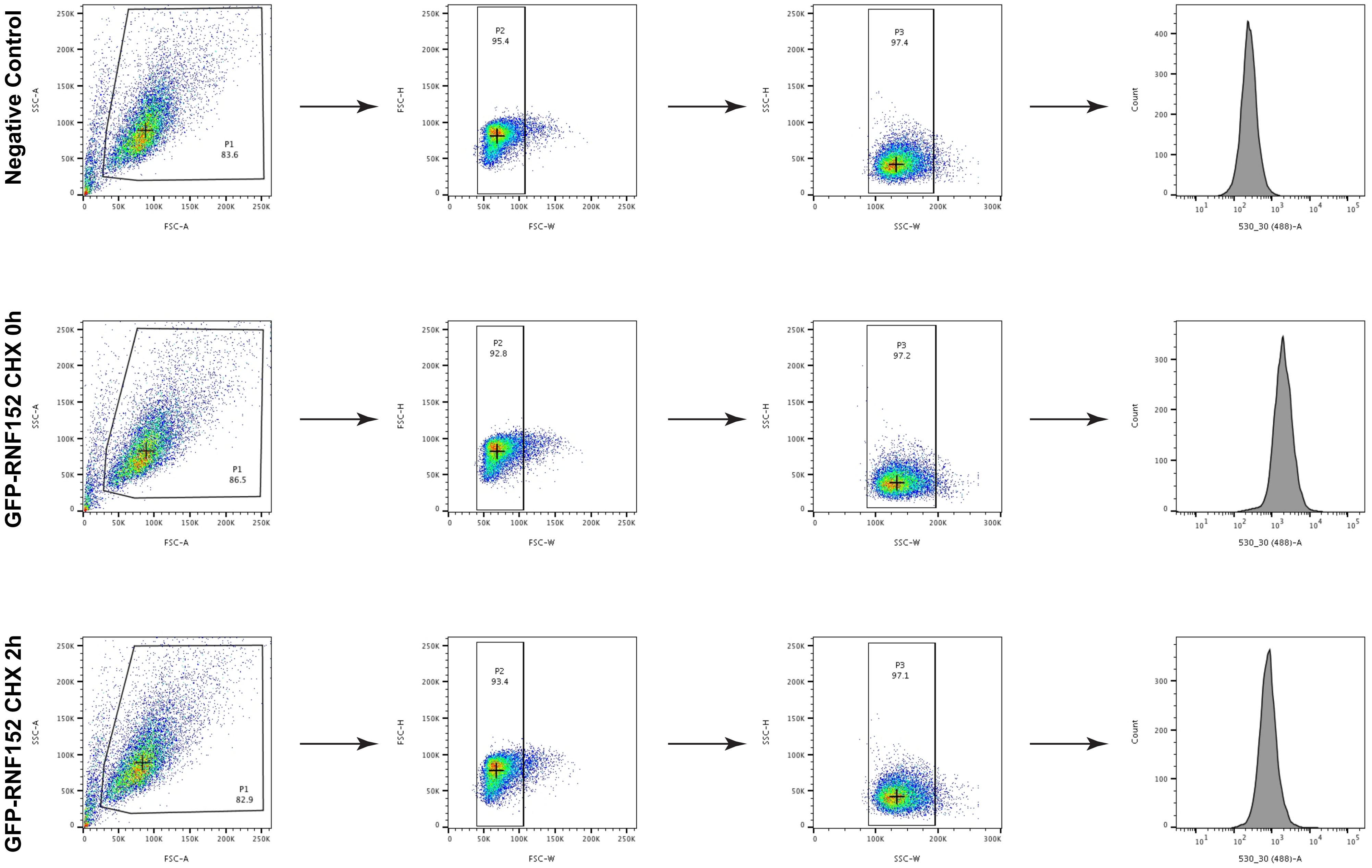

FACS Repository ID:

Figure #	Repository ID	Figure #	Repository ID
2D	FR-FCM-Z45K & L	5B	FR-FCM-Z45N
4E	FR-FCM-Z45M	5H	FR-FCM-Z45X
