# Supplementary material for: A conserved ubiquitin- and ESCRT-dependent pathway internalizes human lysosomal membrane proteins for degradation
Source: PLoS Biol. 2021 Jul 23;19(7):e3001361. doi: 10.1371/journal.pbio.3001361 (PMC8337054; doi:10.1371/journal.pbio.3001361)

Figure 1A

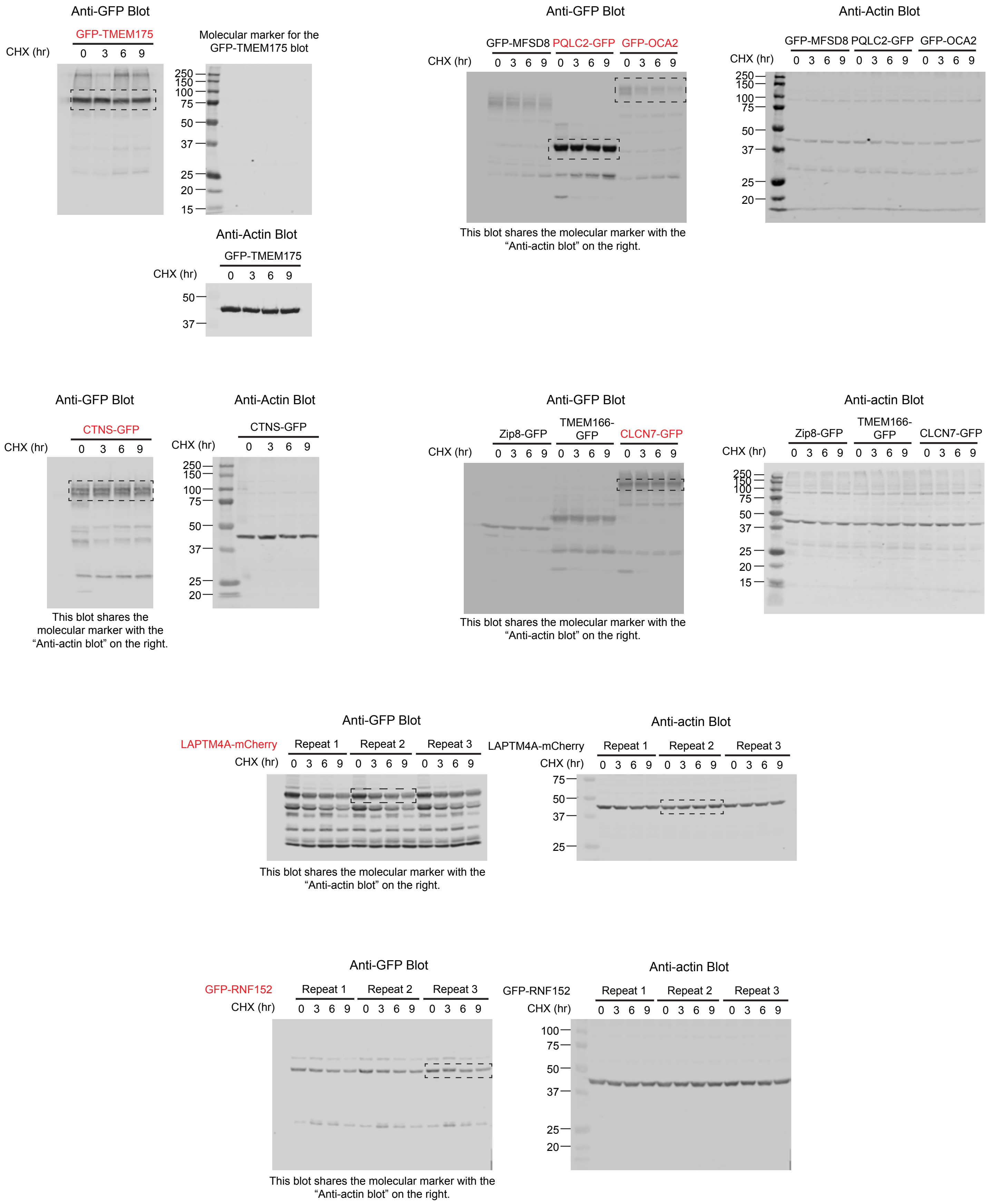
Anti-GFP Blot
GFP-TMEM175
CHX (hr)
0 3 6 9
Molecular marker for the GFP-TMEM175 blot
250
150
100
75
50
37
25
20
15
Anti-Actin Blot
GFP-TMEM175
CHX (hr)
0 3 6 9
50
37
Anti-GFP Blot
GFP-MFSD8 PQLC2-GFP GFP-OCA2
CHX (hr)
0 3 6 9 0 3 6 9 0 3 6 9
This blot shares the molecular marker with the "Anti-actin blot" on the right.
Anti-Actin Blot
GFP-MFSD8 PQLC2-GFP GFP-OCA2
CHX (hr)
0 3 6 9 0 3 6 9 0 3 6 9
250
150
100
75
50
37
25
20
Anti-GFP Blot
CTNS-GFP
CHX (hr)
0 3 6 9
This blot shares the molecular marker with the "Anti-actin blot" on the right.
Anti-Actin Blot
CTNS-GFP
CHX (hr)
0 3 6 9
250
150
100
75
50
37
25
20
Anti-GFP Blot
Zip8-GFP TMEM166-GFP CLCN7-GFP
CHX (hr)
0 3 6 9 0 3 6 9 0 3 6 9
This blot shares the molecular marker with the "Anti-actin blot" on the right.
Anti-actin Blot
Zip8-GFP TMEM166-GFP CLCN7-GFP
CHX (hr)
0 3 6 9 0 3 6 9 0 3 6 9
250
150
100
75
50
37
25
20
15
Anti-GFP Blot
LAPTM4A-mCherry
Repeat 1 Repeat 2 Repeat 3
CHX (hr)
0 3 6 9 0 3 6 9 0 3 6 9
This blot shares the molecular marker with the "Anti-actin blot" on the right.
Anti-actin Blot
LAPTM4A-mCherry
Repeat 1 Repeat 2 Repeat 3
CHX (hr)
0 3 6 9 0 3 6 9 0 3 6 9
75
50
37
25
Anti-GFP Blot
GFP-RNF152
Repeat 1 Repeat 2 Repeat 3
CHX (hr)
0 3 6 9 0 3 6 9 0 3 6 9
This blot shares the molecular marker with the "Anti-actin blot" on the right.
Anti-actin Blot
GFP-RNF152
Repeat 1 Repeat 2 Repeat 3
CHX (hr)
0 3 6 9 0 3 6 9 0 3 6 9
100
75
50
37
25
20

Figure 1C

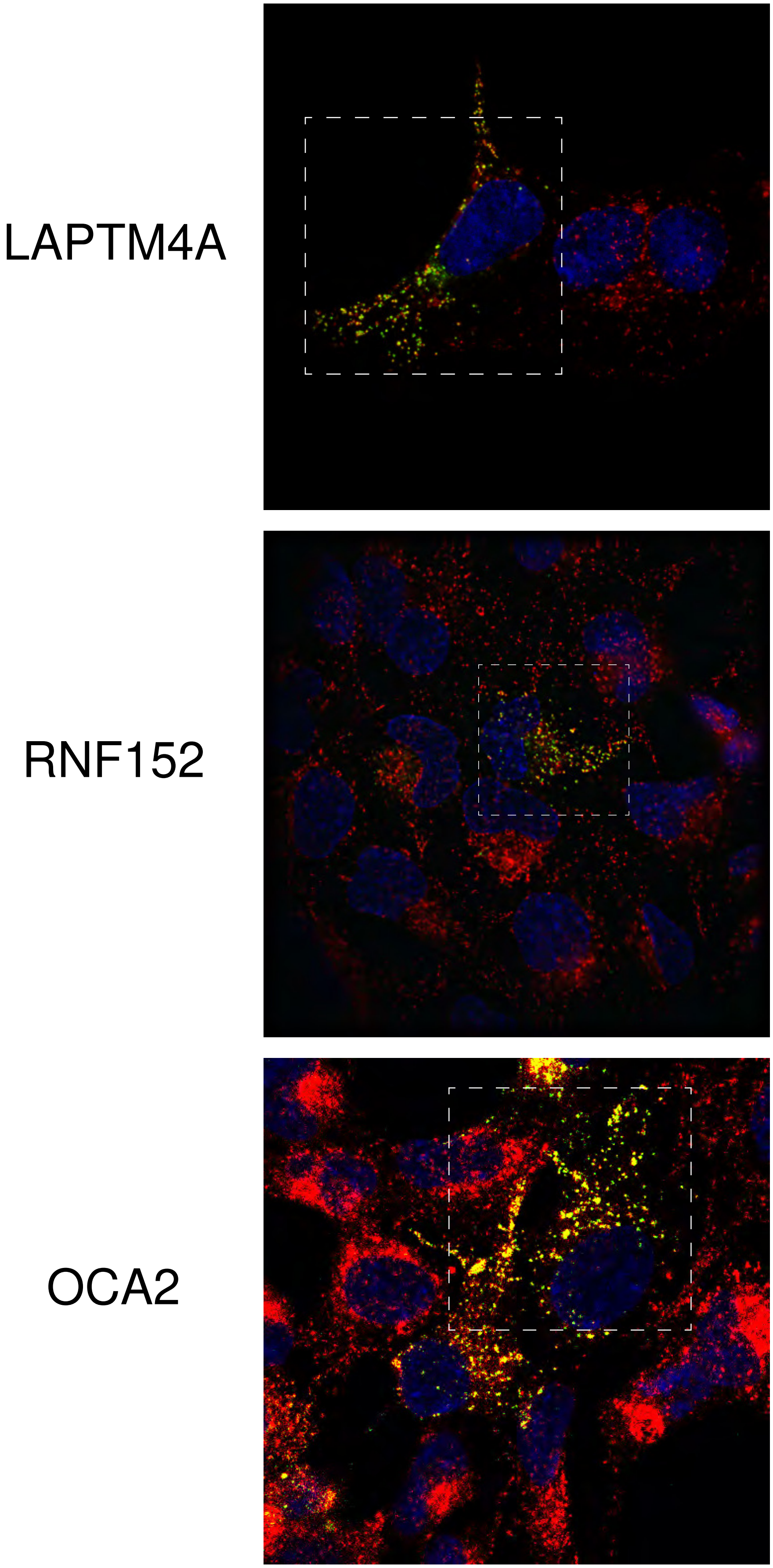

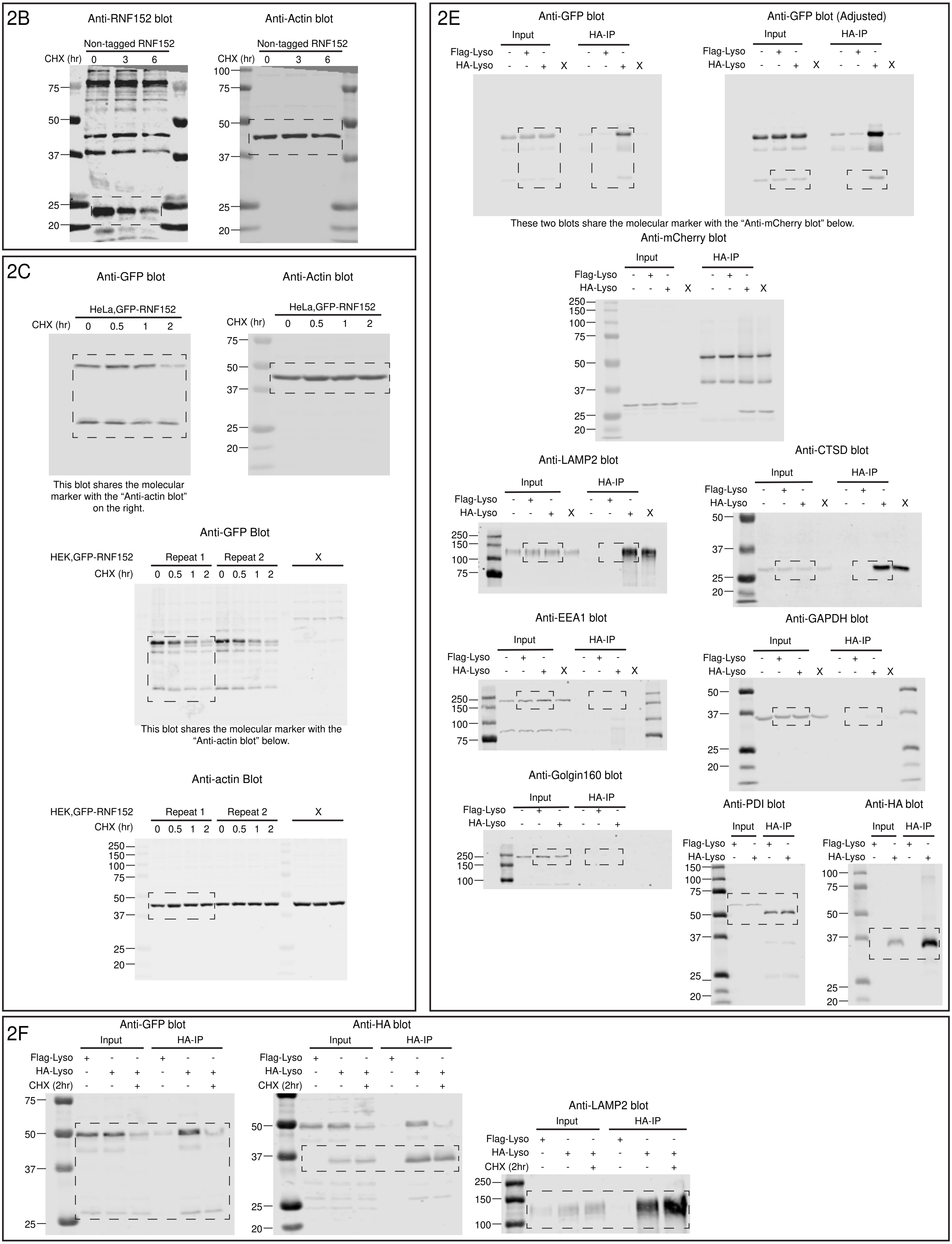
2B
Anti-RNF152 blot
Anti-Actin blot
Non-tagged RNF152
CHX (hr) 0 3 6
2C
Anti-GFP blot
Anti-Actin blot
HeLa,GFP-RNF152
CHX (hr) 0 0.5 1 2
This blot shares the molecular marker with the "Anti-actin blot" on the right.
Anti-GFP Blot
HEK,GFP-RNF152
Repeat 1
Repeat 2
X
This blot shares the molecular marker with the "Anti-actin blot" below.
Anti-actin Blot
2E
Anti-GFP blot
Anti-GFP blot (Adjusted)
Input
HA-IP
Flag-Lyso
HA-Lyso
These two blots share the molecular marker with the "Anti-mCherry blot" below.
Anti-mCherry blot
Anti-LAMP2 blot
Anti-CTSD blot
Anti-EEA1 blot
Anti-GAPDH blot
Anti-Golgin160 blot
Anti-PDI blot
Anti-HA blot
2F
Anti-GFP blot
Anti-HA blot
Anti-LAMP2 blot
CHX (2hr)

Figure 3

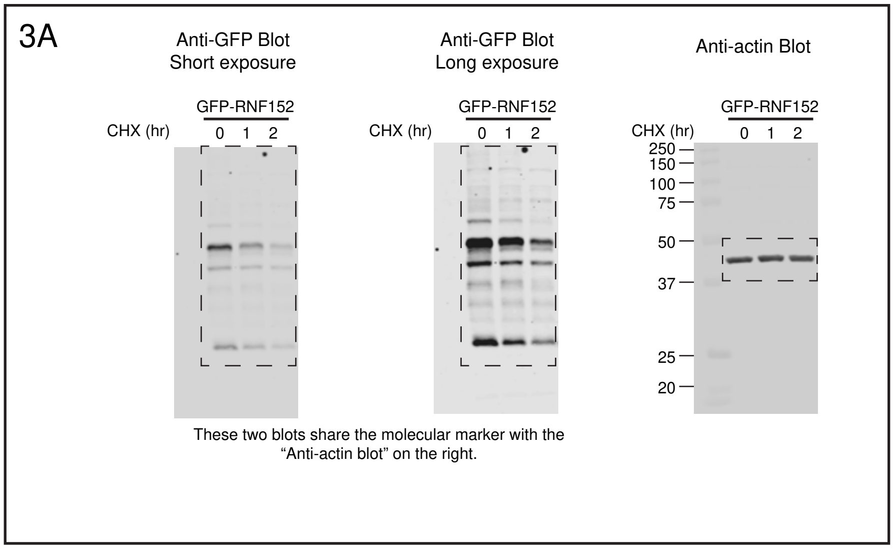
3A
Anti-GFP Blot
Short exposure
Anti-GFP Blot
Long exposure
Anti-actin Blot
GFP-RNF152
CHX (hr)
0 1 2
250
150
100
75
50
37
25
20
These two blots share the molecular marker with the "Anti-actin blot" on the right.

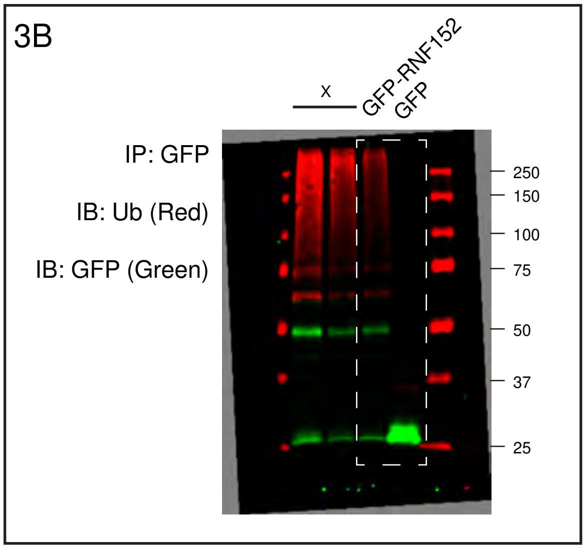
3B
x
GFP-RNF152
GFP
IP: GFP
IB: Ub (Red)
IB: GFP (Green)
250
150
100
75
50
37
25

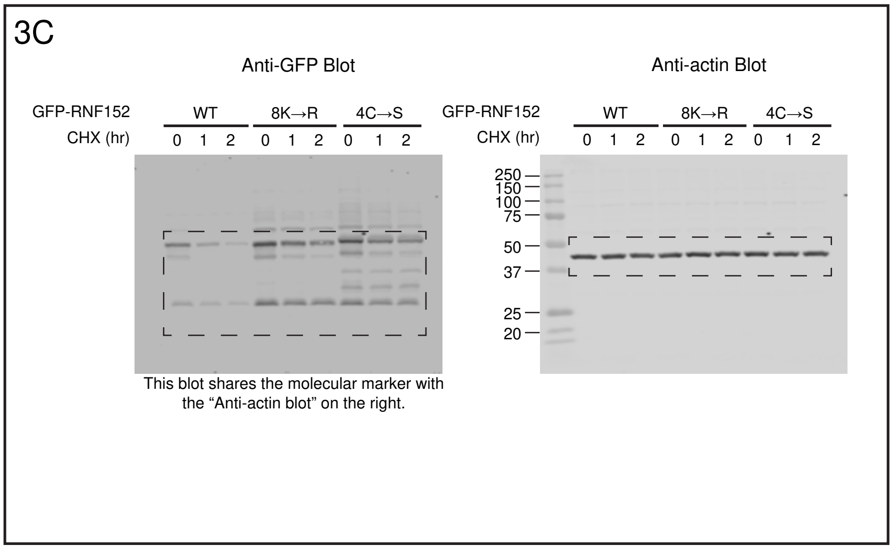
3C
Anti-GFP Blot
Anti-actin Blot
GFP-RNF152
CHX (hr)
WT
8K→R
4C→S
0 1 2 0 1 2 0 1 2
250
150
100
75
50
37
25
20
This blot shares the molecular marker with the "Anti-actin blot" on the right.

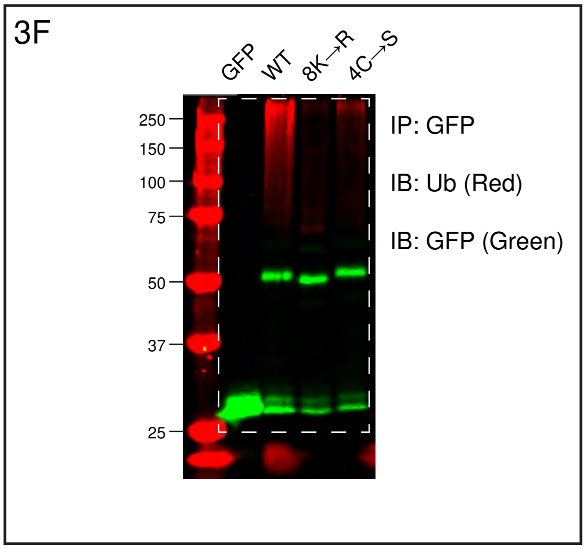
3F
GFP
WT
8K→R
4C→S
IP: GFP
IB: Ub (Red)
IB: GFP (Green)
250
150
100
75
50
37
25

Figure 4

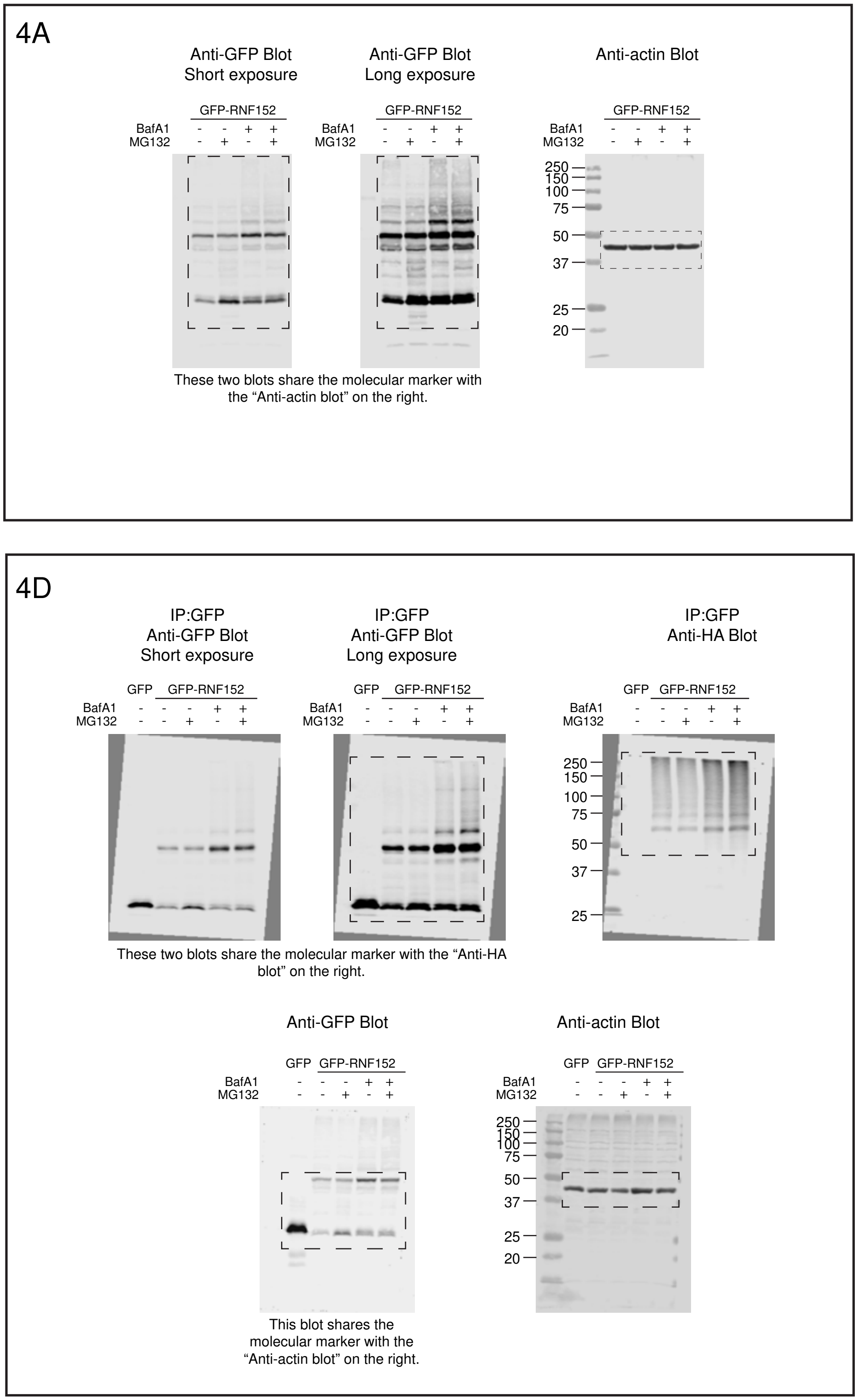
4A
Anti-GFP Blot
Short exposure
Anti-GFP Blot
Long exposure
Anti-actin Blot
GFP-RNF152
BafA1 - - + +
MG132 - + - +
250
150
100
75
50
37
25
20
These two blots share the molecular marker with the "Anti-actin blot" on the right.
4D
IP:GFP
Anti-GFP Blot
Short exposure
IP:GFP
Anti-GFP Blot
Long exposure
IP:GFP
Anti-HA Blot
GFP
GFP-RNF152
BafA1 - - - + +
MG132 - - + - +
250
150
100
75
50
37
25
These two blots share the molecular marker with the "Anti-HA blot" on the right.
Anti-GFP Blot
Anti-actin Blot
250
150
100
75
50
37
25
20
This blot shares the molecular marker with the "Anti-actin blot" on the right.

Figure 4F-G

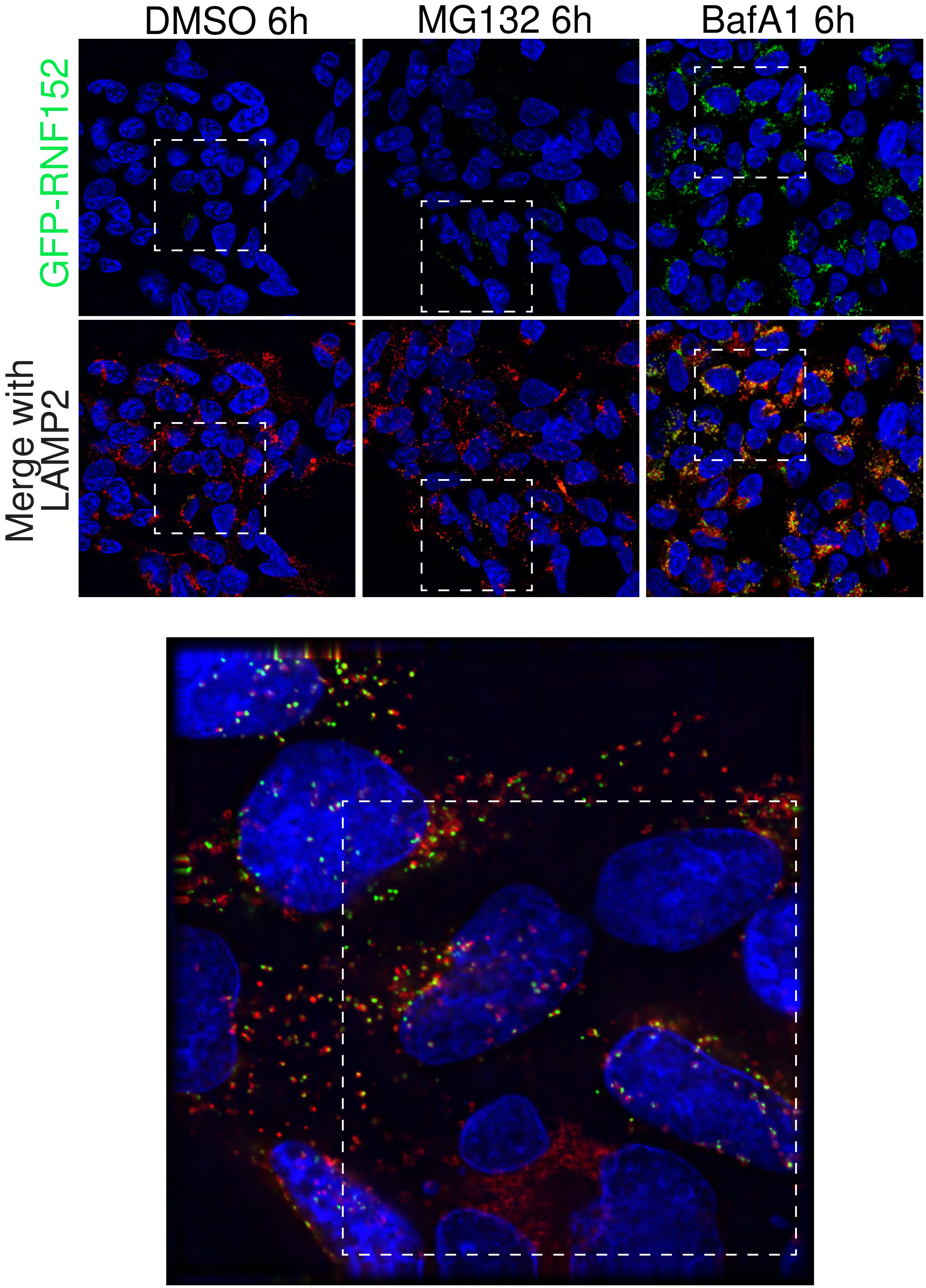

Figure 5

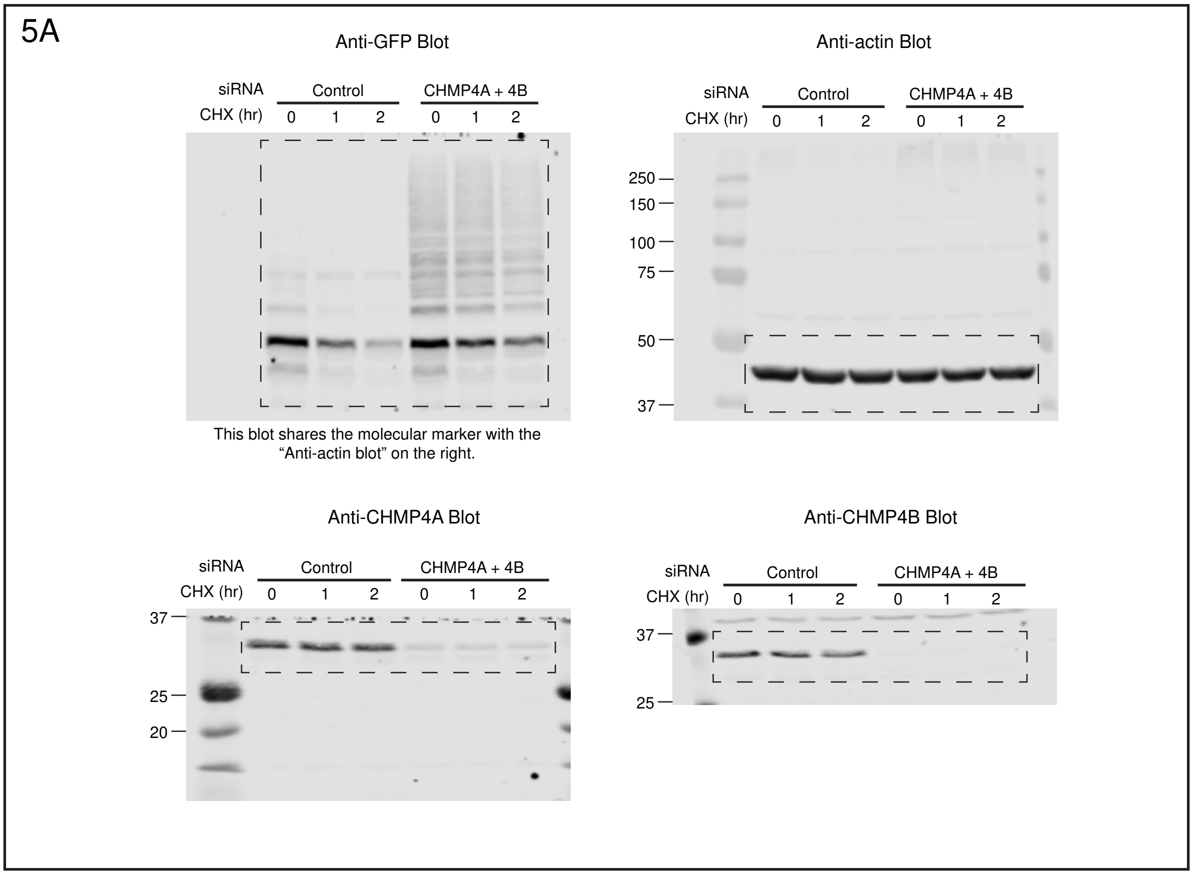

5A
Anti-GFP Blot
siRNA
Control
CHMP4A + 4B
CHX (hr)
0 1 2 0 1 2
This blot shares the molecular marker with the "Anti-actin blot" on the right.
Anti-actin Blot
siRNA
Control
CHMP4A + 4B
CHX (hr)
0 1 2 0 1 2
250
150
100
75
50
37
Anti-CHMP4A Blot
siRNA
Control
CHMP4A + 4B
CHX (hr)
0 1 2 0 1 2
37
25
20
Anti-CHMP4B Blot
siRNA
Control
CHMP4A + 4B
CHX (hr)
0 1 2 0 1 2
37
25

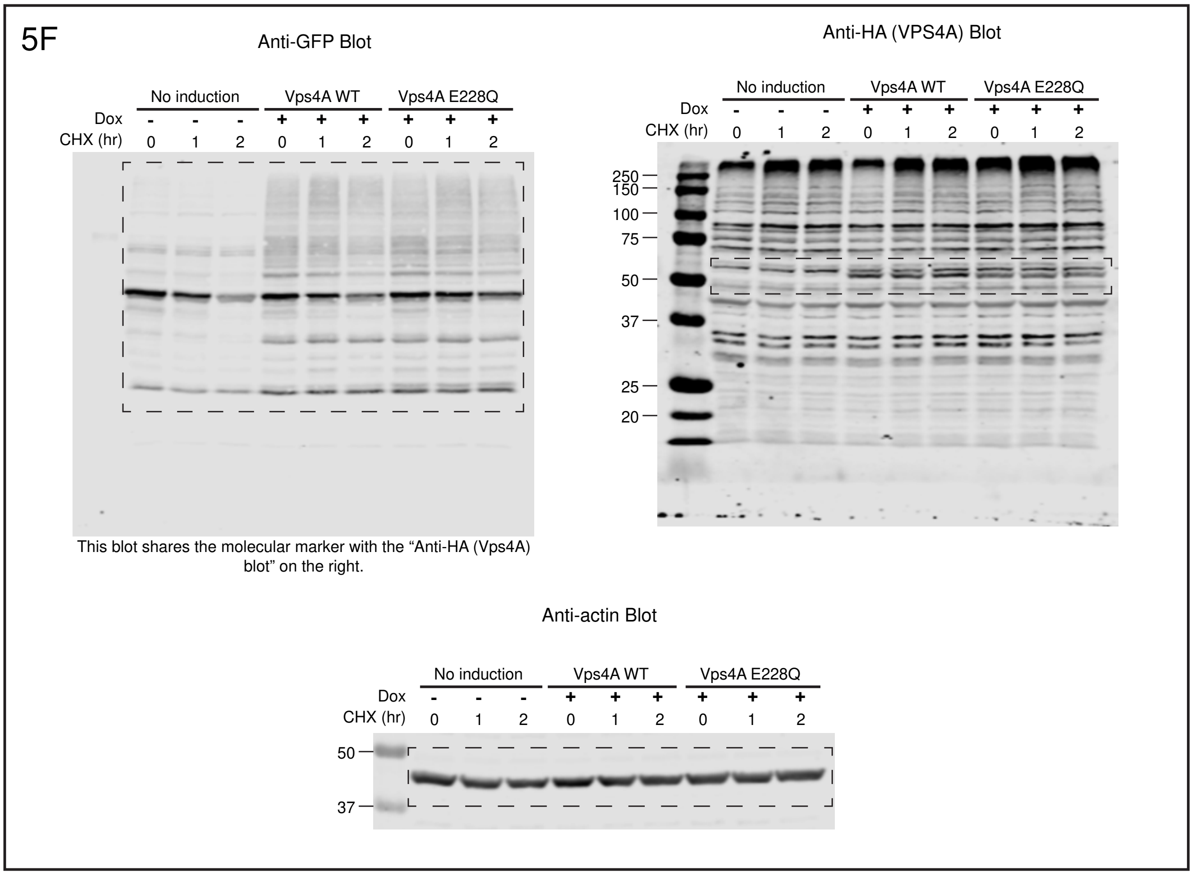

5F
Anti-GFP Blot
No induction
Vps4A WT
Vps4A E228Q
Dox
- - - + + + + + +
CHX (hr)
0 1 2 0 1 2 0 1 2
This blot shares the molecular marker with the "Anti-HA (Vps4A) blot" on the right.
Anti-HA (VPS4A) Blot
No induction
Vps4A WT
Vps4A E228Q
Dox
- - - + + + + + +
CHX (hr)
0 1 2 0 1 2 0 1 2
250
150
100
75
50
37
25
20
Anti-actin Blot
No induction
Vps4A WT
Vps4A E228Q
Dox
- - - + + + + + +
CHX (hr)
0 1 2 0 1 2 0 1 2
50
37

Figure 5D

si Control

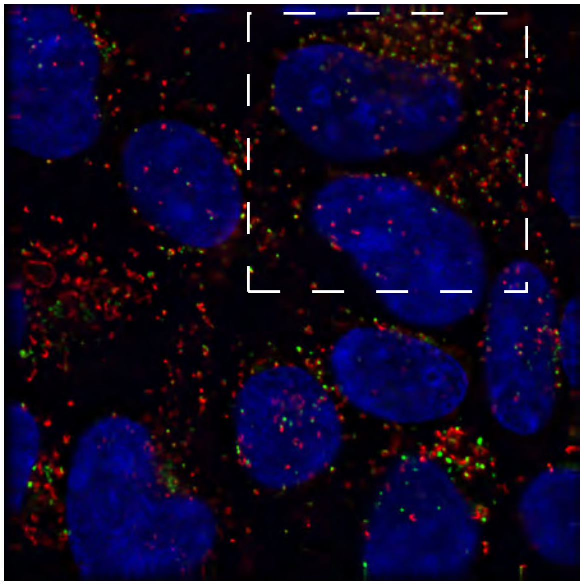

si CHMP4A+4B

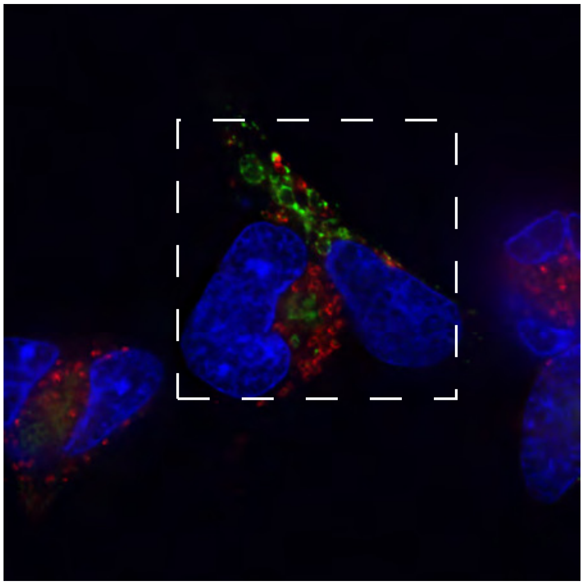

Figure 6

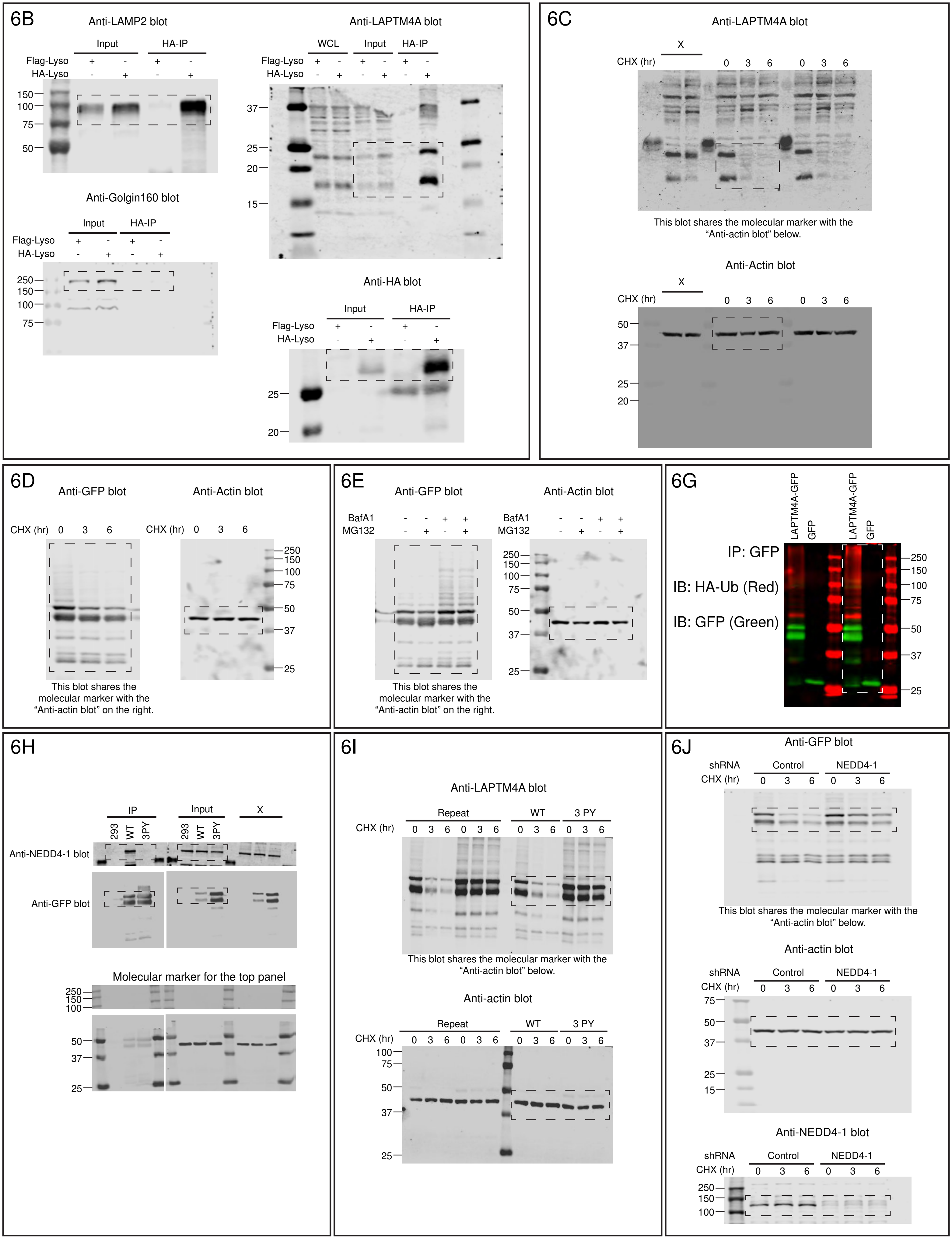
6B
Anti-LAMP2 blot
Input
HA-IP
Flag-Lyso + - + -
HA-Lyso - + - +
150
100
75
50
Anti-Golgin160 blot
Input
HA-IP
Flag-Lyso + - + -
HA-Lyso - + - +
250
150
100
75
Anti-LAPTM4A blot
WCL
Input
HA-IP
Flag-Lyso + - + - + -
HA-Lyso - + - + - +
37
25
20
15
Anti-HA blot
Input
HA-IP
Flag-Lyso + - + -
HA-Lyso - + - +
25
20
6C
Anti-LAPTM4A blot
X
CHX (hr) 0 3 6 0 3 6
This blot shares the molecular marker with the "Anti-actin blot" below.
Anti-Actin blot
X
CHX (hr) 0 3 6 0 3 6
50
37
25
20
6D
Anti-GFP blot
CHX (hr) 0 3 6
This blot shares the molecular marker with the "Anti-actin blot" on the right.
Anti-Actin blot
CHX (hr) 0 3 6
250
150
100
75
50
37
25
6E
Anti-GFP blot
BafA1 - - + +
MG132 - + - +
This blot shares the molecular marker with the "Anti-actin blot" on the right.
Anti-Actin blot
BafA1 - - + +
MG132 - + - +
250
150
100
75
50
37
25
6G
LAPTM4A-GFP
GFP
LAPTM4A-GFP
GFP
IP: GFP
IB: HA-Ub (Red)
IB: GFP (Green)
250
150
100
75
50
37
25
6H
IP
Input
X
293 WT 3PY 293 WT 3PY
Anti-NEDD4-1 blot
Anti-GFP blot
Molecular marker for the top panel
250
150
100
50
37
25
6I
Anti-LAPTM4A blot
Repeat
WT
3 PY
CHX (hr) 0 3 6 0 3 6 0 3 6 0 3 6
This blot shares the molecular marker with the "Anti-actin blot" below.
Anti-actin blot
Repeat
WT
3 PY
CHX (hr) 0 3 6 0 3 6 0 3 6 0 3 6
100
75
50
37
25
6J
Anti-GFP blot
shRNA Control NEDD4-1
CHX (hr) 0 3 6 0 3 6
This blot shares the molecular marker with the "Anti-actin blot" below.
Anti-actin blot
shRNA Control NEDD4-1
CHX (hr) 0 3 6 0 3 6
75
50
37
25
15
Anti-NEDD4-1 blot
shRNA Control NEDD4-1
CHX (hr) 0 3 6 0 3 6
250
150
100

Figure 7

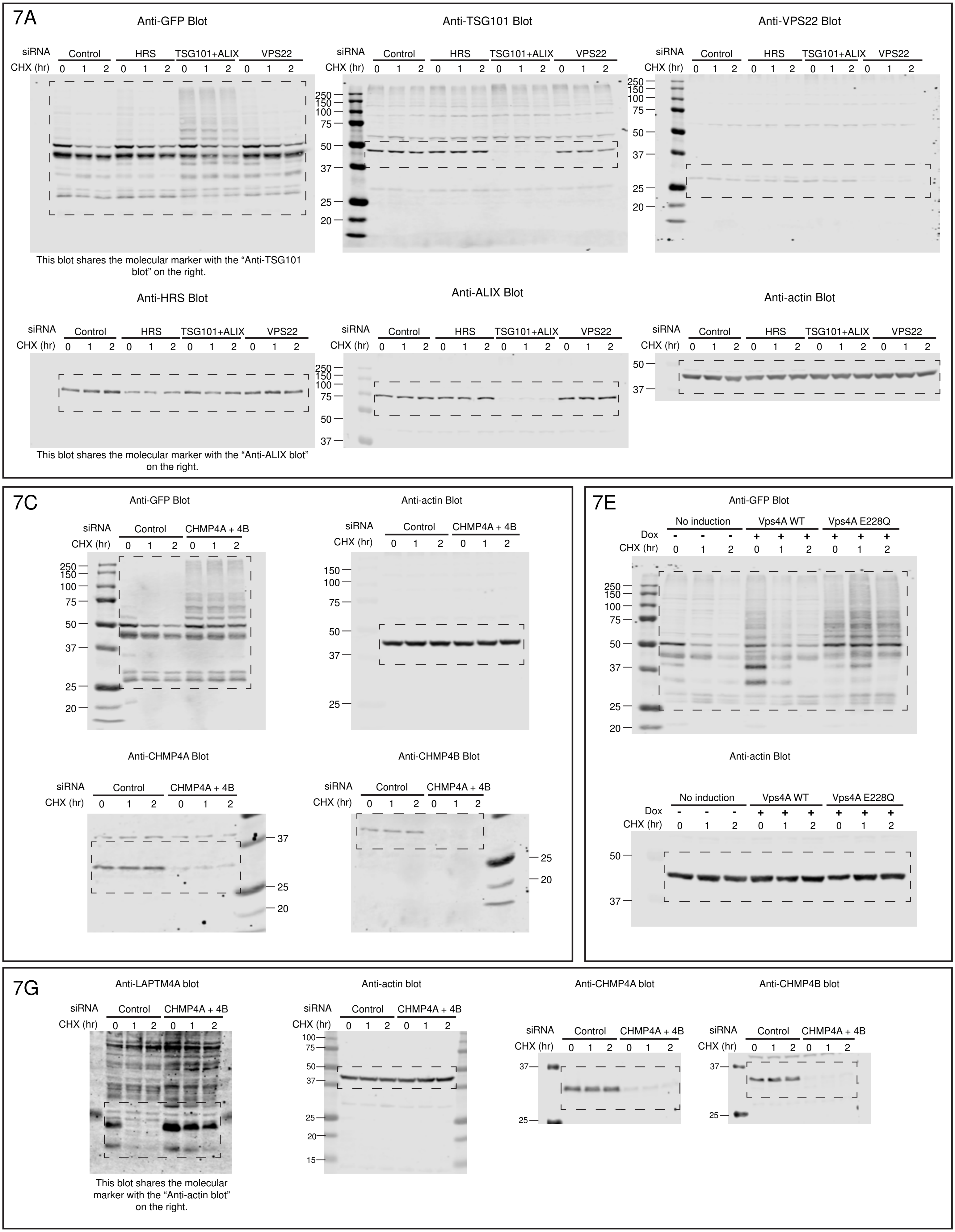
7A
Anti-GFP Blot
Anti-TSG101 Blot
Anti-VPS22 Blot
siRNA
Control
HRS
TSG101+ALIX
VPS22
CHX (hr)
0 1 2 0 1 2 0 1 2 0 1 2
250
150
100
75
50
37
25
20
This blot shares the molecular marker with the "Anti-TSG101 blot" on the right.
Anti-HRS Blot
Anti-ALIX Blot
Anti-actin Blot
This blot shares the molecular marker with the "Anti-ALIX blot" on the right.
7C
Anti-GFP Blot
Anti-actin Blot
siRNA
Control
CHMP4A + 4B
CHX (hr)
0 1 2 0 1 2
Anti-CHMP4A Blot
Anti-CHMP4B Blot
7E
Anti-GFP Blot
No induction
Vps4A WT
Vps4A E228Q
Dox
- - - + + + + + +
CHX (hr)
0 1 2 0 1 2 0 1 2
Anti-actin Blot
7G
Anti-LAPTM4A blot
Anti-actin blot
Anti-CHMP4A blot
Anti-CHMP4B blot
15
This blot shares the molecular marker with the "Anti-actin blot" on the right.

Figure 8A

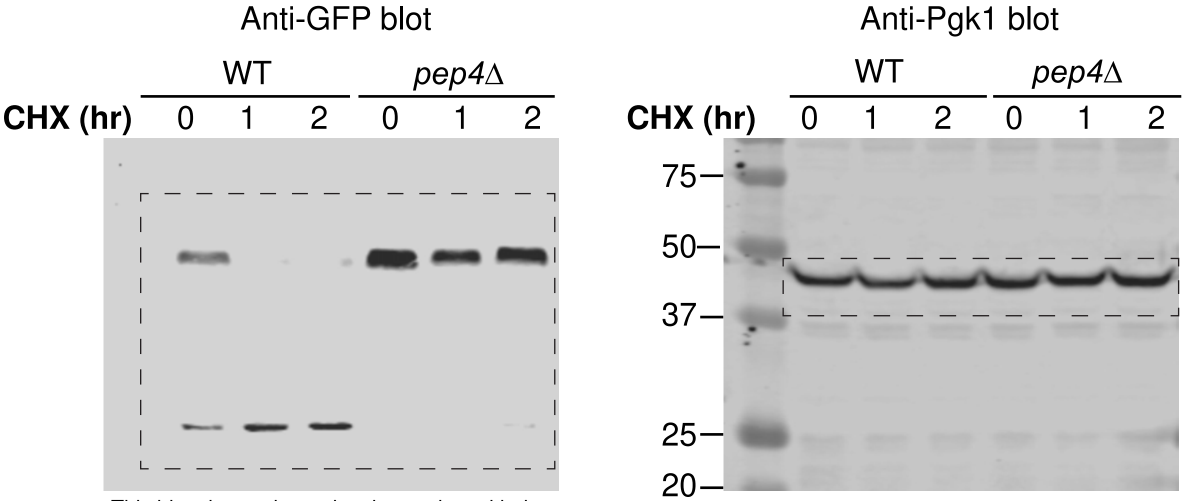

This blot shares the molecular marker with the “Anti-Pgk1 blot” on the right.

Figure 8C-1

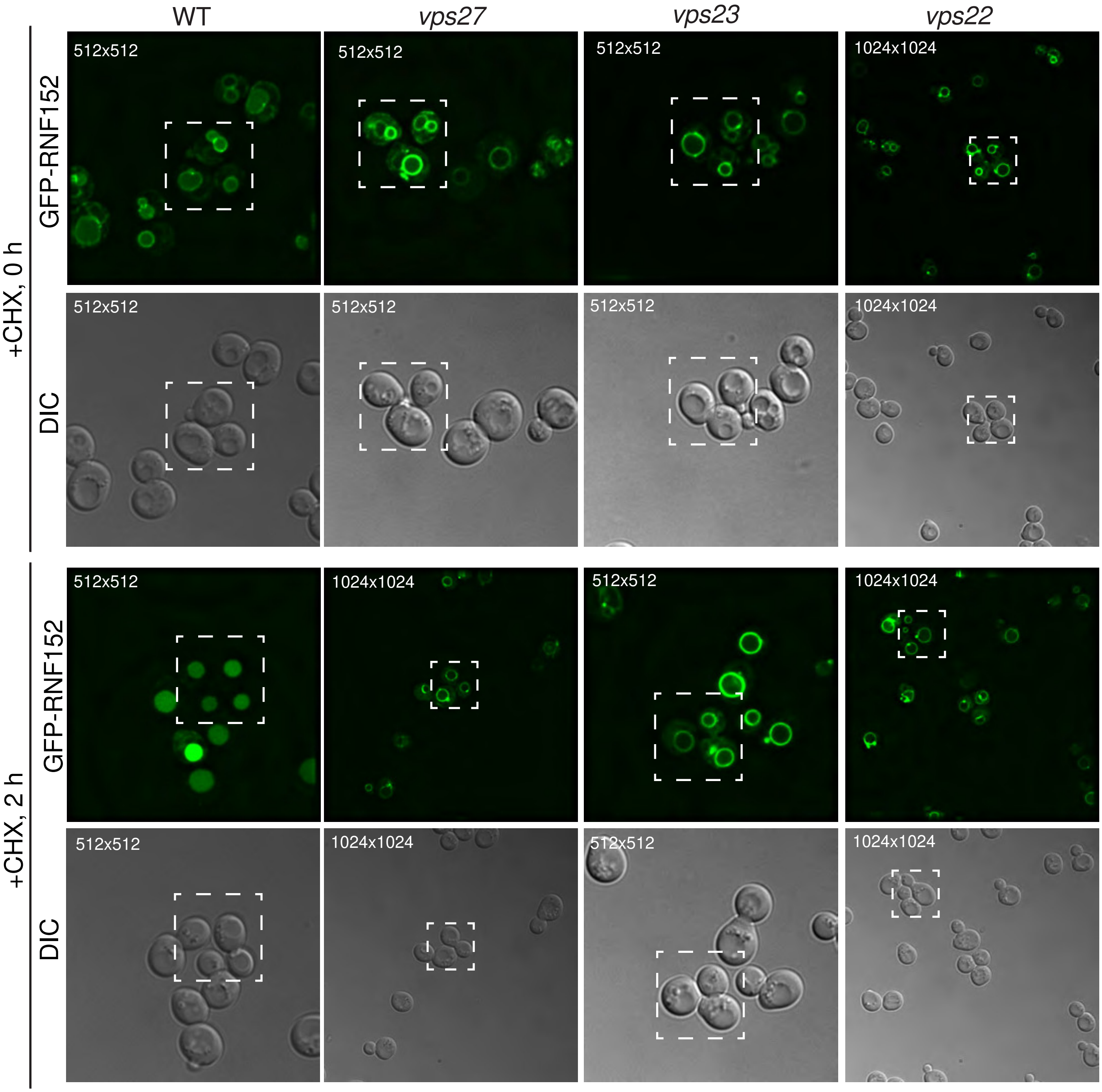

WT
vps27
vps23
vps22
+CHX, 0 h
+CHX, 2 h
GFP-RNF152
DIC
GFP-RNF152
DIC
512x512
1024x1024

Figure 8C-2

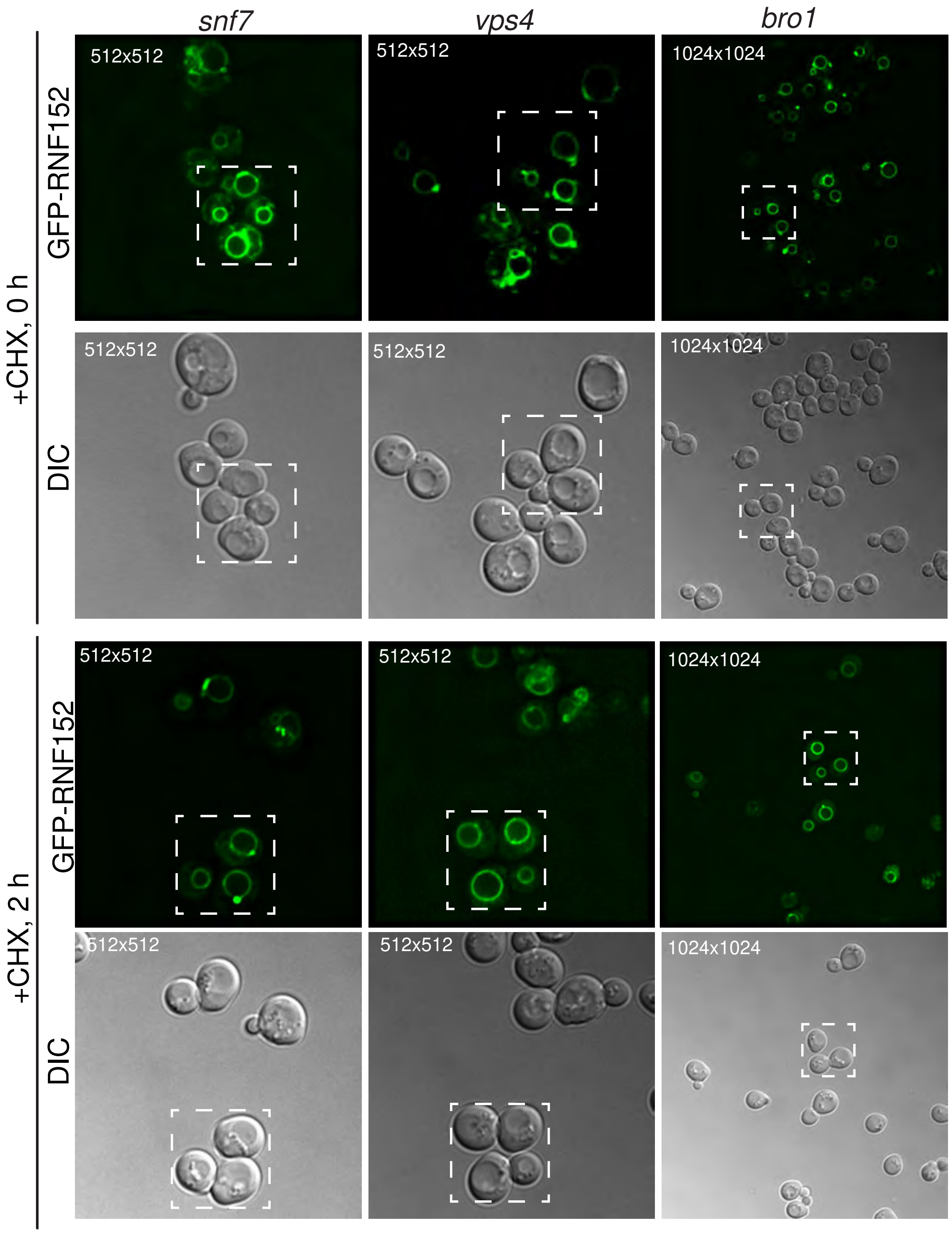

snf7
vps4
bro1
+CHX, 0 h
GFP-RNF152
DIC
+CHX, 2 h
GFP-RNF152
DIC
512x512
1024x1024

Figure S1-1

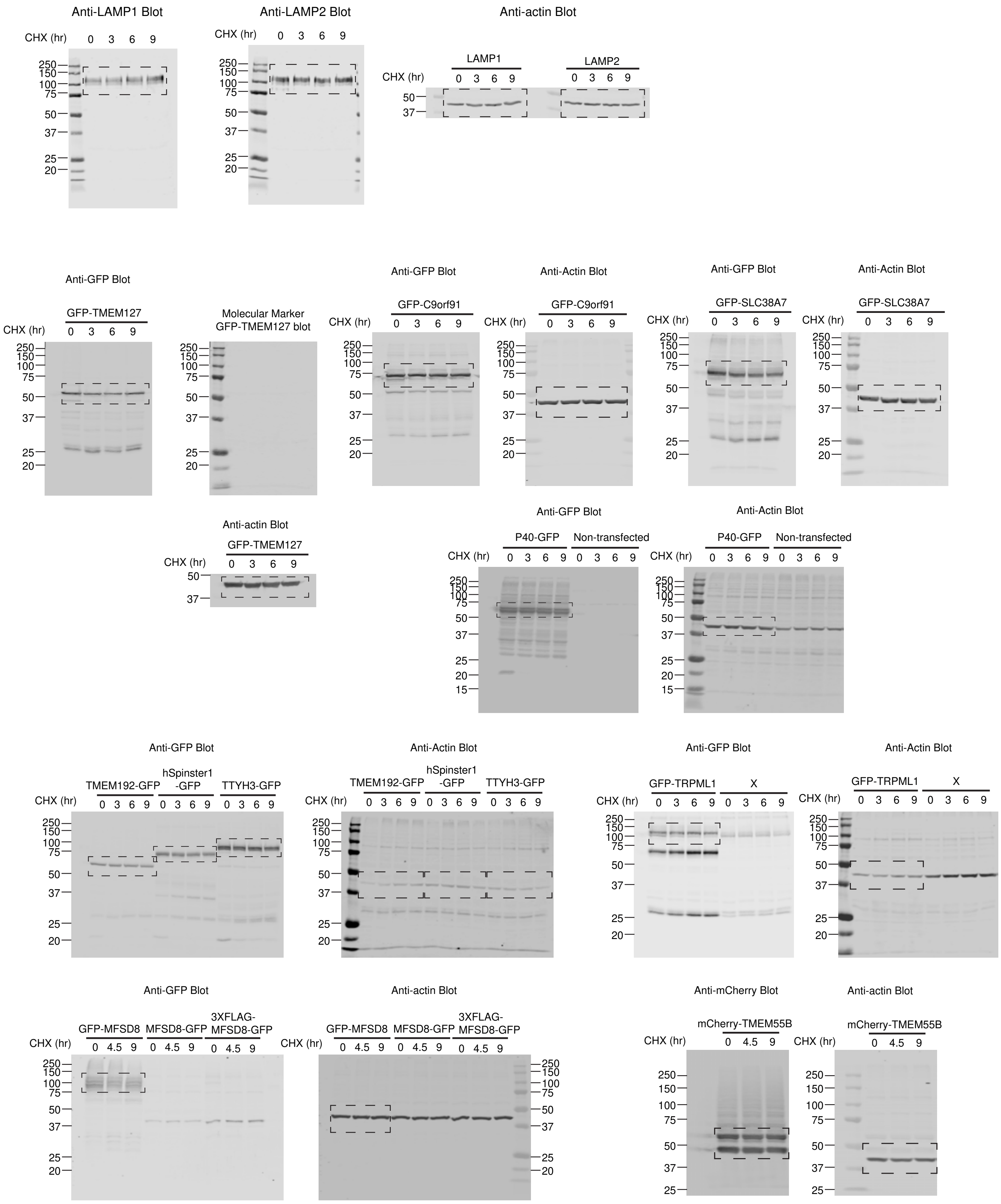
Anti-LAMP1 Blot
CHX (hr) 0 3 6 9
Anti-LAMP2 Blot
CHX (hr) 0 3 6 9
Anti-actin Blot
LAMP1
LAMP2
CHX (hr) 0 3 6 9 0 3 6 9
250 150 100 75 50 37 25 20
Anti-GFP Blot
GFP-TMEM127
Molecular Marker
GFP-TMEM127 blot
Anti-GFP Blot
GFP-C9orf91
Anti-Actin Blot
GFP-C9orf91
Anti-GFP Blot
GFP-SLC38A7
Anti-Actin Blot
GFP-SLC38A7
Anti-actin Blot
GFP-TMEM127
Anti-GFP Blot
P40-GFP
Non-transfected
Anti-Actin Blot
P40-GFP
Non-transfected
15
Anti-GFP Blot
TMEM192-GFP
hSpinster1
-GFP
TTYH3-GFP
Anti-Actin Blot
TMEM192-GFP
hSpinster1
-GFP
TTYH3-GFP
Anti-GFP Blot
GFP-TRPML1
X
Anti-Actin Blot
GFP-TRPML1
X
Anti-GFP Blot
GFP-MFSD8
MFSD8-GFP
3XFLAG-
MFSD8-GFP
CHX (hr) 0 4.5 9 0 4.5 9 0 4.5 9
Anti-actin Blot
GFP-MFSD8
MFSD8-GFP
3XFLAG-
MFSD8-GFP
Anti-mCherry Blot
mCherry-TMEM55B
CHX (hr) 0 4.5 9
Anti-actin Blot
mCherry-TMEM55B

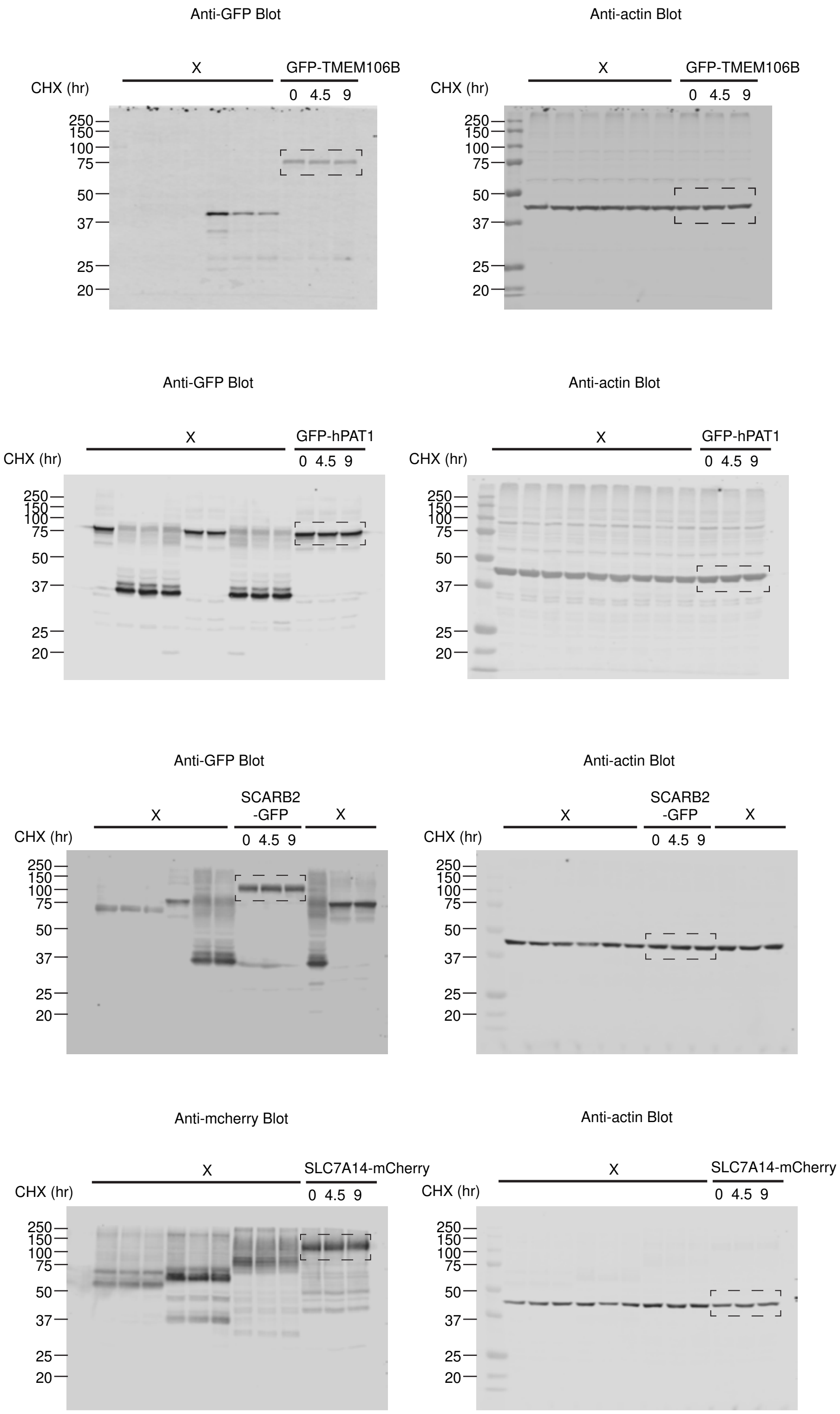

Anti-GFP Blot
Anti-actin Blot
X
GFP-TMEM106B
CHX (hr)
0 4.5 9
250
150
100
75
50
37
25
20
Anti-GFP Blot
Anti-actin Blot
X
GFP-hPAT1
CHX (hr)
0 4.5 9
Anti-GFP Blot
Anti-actin Blot
X
SCARB2
-GFP
X
CHX (hr)
0 4.5 9
Anti-mcherry Blot
Anti-actin Blot
X
SLC7A14-mCherry
CHX (hr)
0 4.5 9

Figure S2

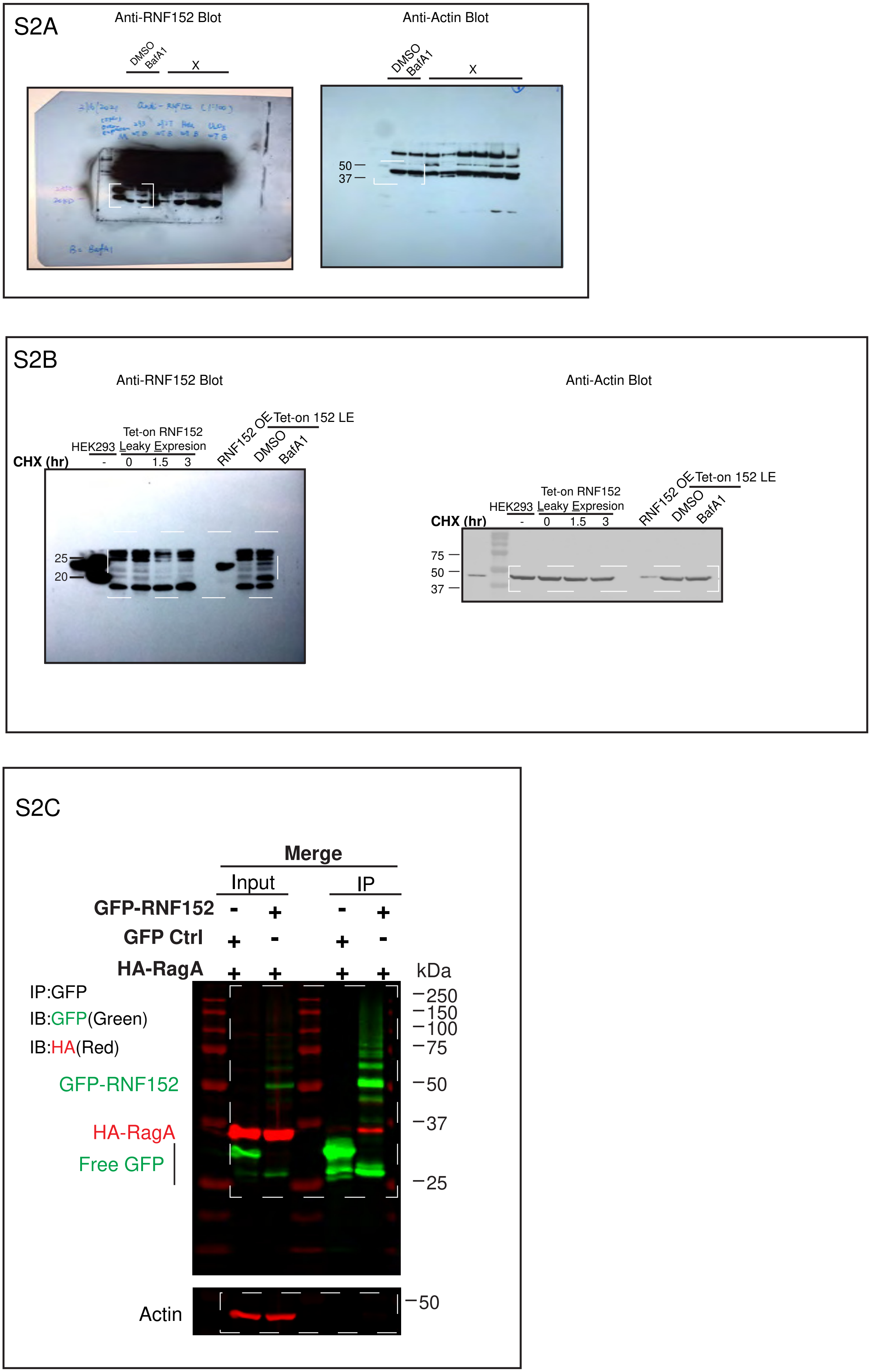
S2A
Anti-RNF152 Blot
DMSO
BafA1
X
Anti-Actin Blot
DMSO
BafA1
X
50
37
S2B
Anti-RNF152 Blot
CHX (hr)
HEK293
Tet-on RNF152 Leaky Expresion
-
0
1.5
3
RNF152 OE
Tet-on 152 LE
DMSO
BafA1
25
20
Anti-Actin Blot
CHX (hr)
HEK293
Tet-on RNF152 Leaky Expresion
-
0
1.5
3
RNF152 OE
Tet-on 152 LE
DMSO
BafA1
75
50
37
S2C
Merge
Input
IP
GFP-RNF152 - + - +
GFP Ctrl + - + -
HA-RagA + + + +
kDa
IP:GFP
IB:GFP(Green)
IB:HA(Red)
GFP-RNF152
HA-RagA
Free GFP
250
150
100
75
50
37
25
Actin
50

Figure S3

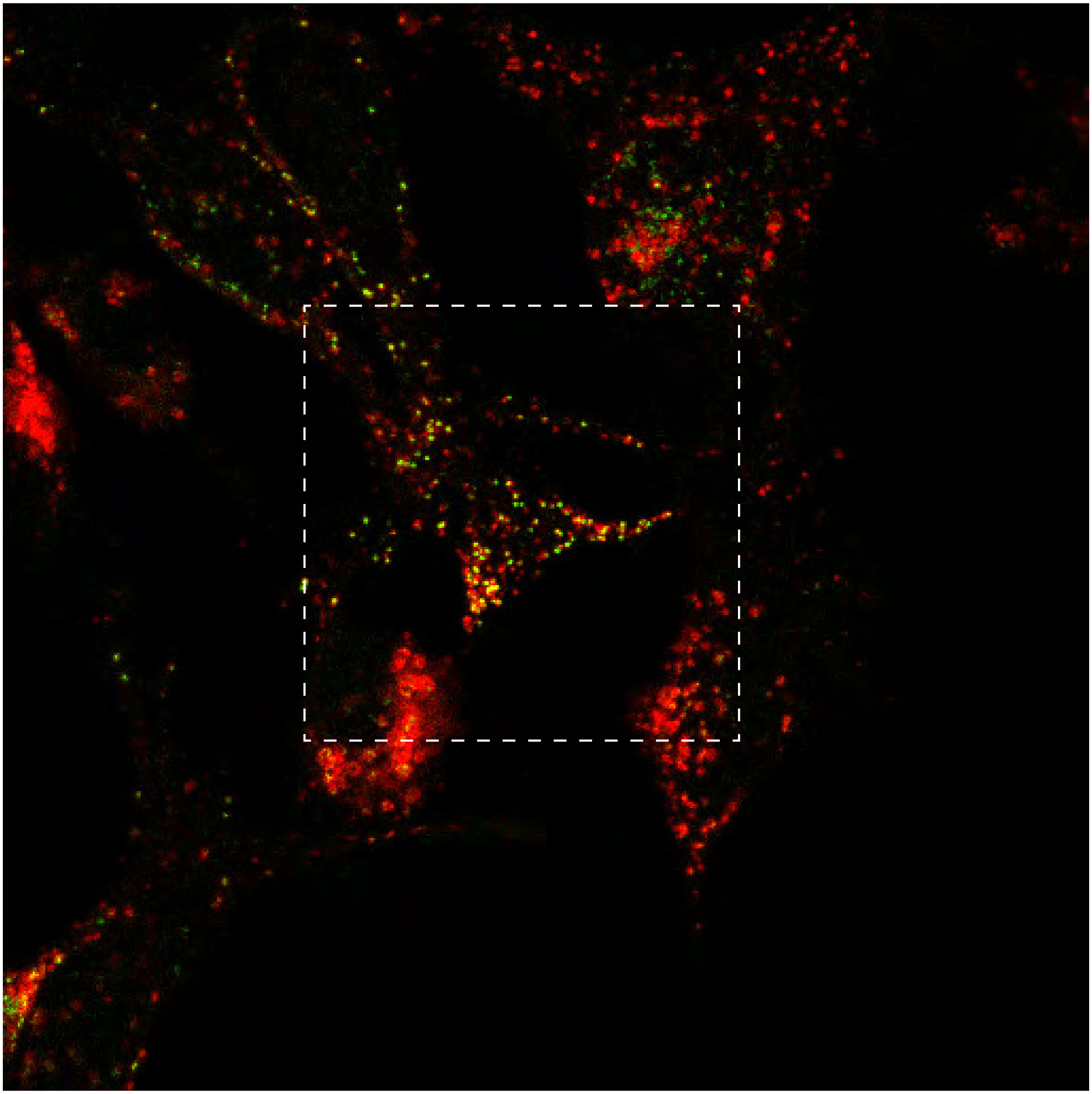

Figure S4A

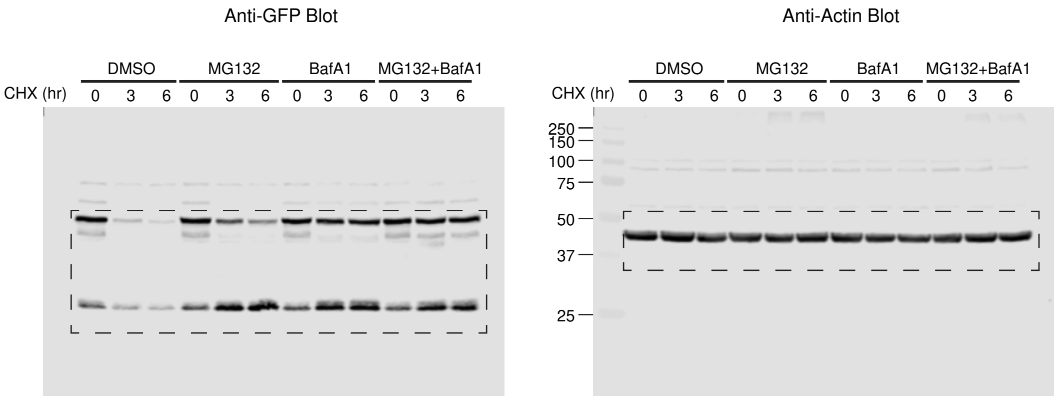
Anti-GFP Blot
DMSO
MG132
BafA1
MG132+BafA1
CHX (hr)
0 3 6 0 3 6 0 3 6 0 3 6
Anti-Actin Blot
DMSO
MG132
BafA1
MG132+BafA1
CHX (hr)
0 3 6 0 3 6 0 3 6 0 3 6
250
150
100
75
50
37
25

Figure S5

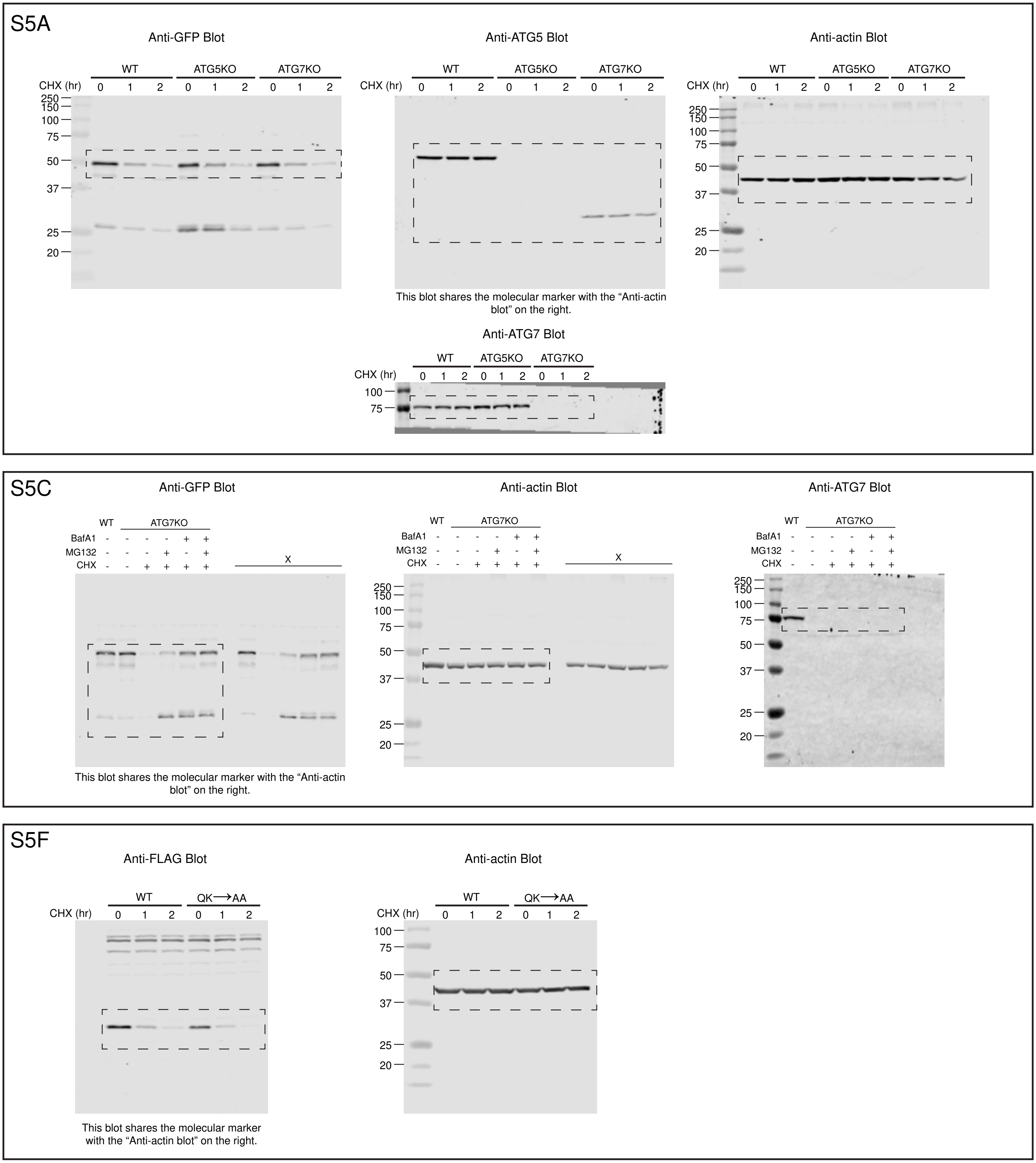
S5A
Anti-GFP Blot
WT
ATG5KO
ATG7KO
CHX (hr)
0 1 2 0 1 2 0 1 2
250
150
100
75
50
37
25
20
Anti-ATG5 Blot
This blot shares the molecular marker with the “Anti-actin blot” on the right.
Anti-actin Blot
Anti-ATG7 Blot
S5C
Anti-GFP Blot
BafA1
MG132
CHX
X
This blot shares the molecular marker with the “Anti-actin blot” on the right.
Anti-actin Blot
Anti-ATG7 Blot
S5F
Anti-FLAG Blot
QK⟶AA
This blot shares the molecular marker with the “Anti-actin blot” on the right.
Anti-actin Blot

Figure S6

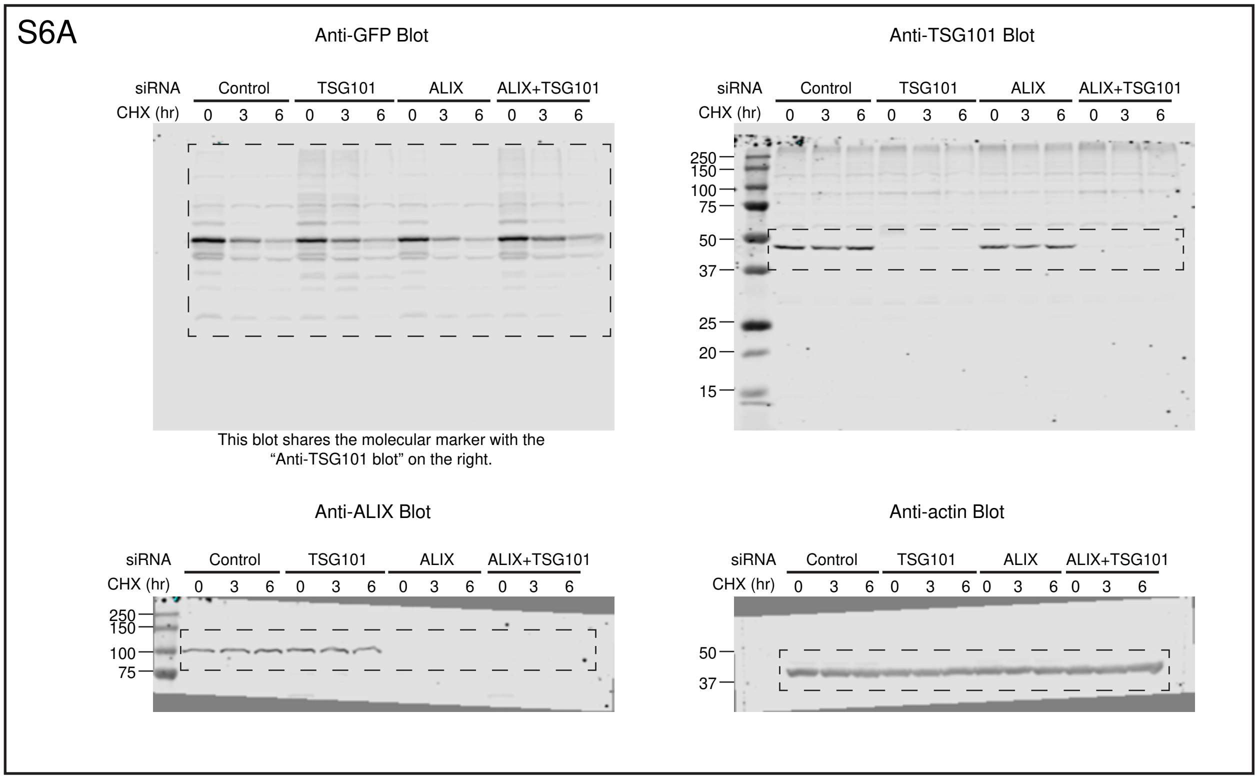
S6A
Anti-GFP Blot
siRNA Control TSG101 ALIX ALIX+TSG101
CHX (hr) 0 3 6 0 3 6 0 3 6 0 3 6
This blot shares the molecular marker with the "Anti-TSG101 blot" on the right.
Anti-TSG101 Blot
siRNA Control TSG101 ALIX ALIX+TSG101
CHX (hr) 0 3 6 0 3 6 0 3 6 0 3 6
250
150
100
75
50
37
25
20
15
Anti-ALIX Blot
siRNA Control TSG101 ALIX ALIX+TSG101
CHX (hr) 0 3 6 0 3 6 0 3 6 0 3 6
250
150
100
75
Anti-actin Blot
siRNA Control TSG101 ALIX ALIX+TSG101
CHX (hr) 0 3 6 0 3 6 0 3 6 0 3 6
50
37

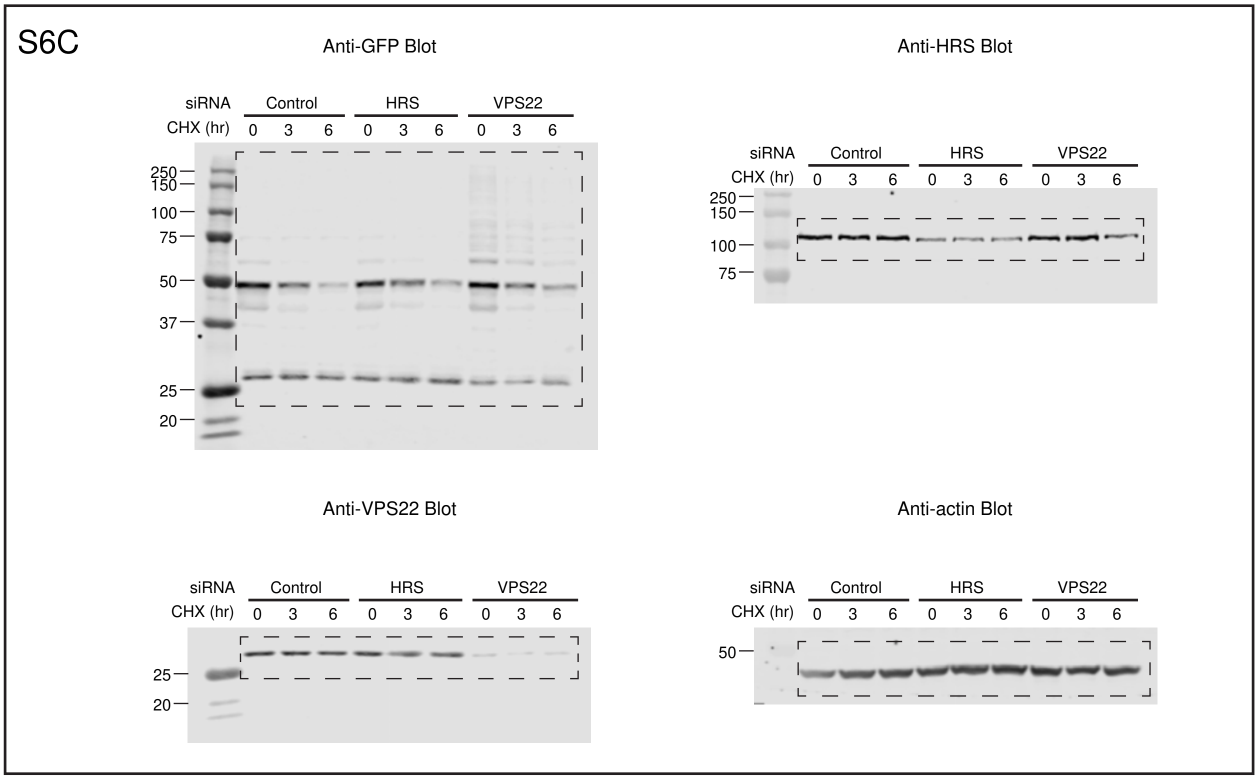
S6C
Anti-GFP Blot
siRNA Control HRS VPS22
CHX (hr) 0 3 6 0 3 6 0 3 6
250
150
100
75
50
37
25
20
Anti-HRS Blot
siRNA Control HRS VPS22
CHX (hr) 0 3 6 0 3 6 0 3 6
250
150
100
75
Anti-VPS22 Blot
siRNA Control HRS VPS22
CHX (hr) 0 3 6 0 3 6 0 3 6
25
20
Anti-actin Blot
siRNA Control HRS VPS22
CHX (hr) 0 3 6 0 3 6 0 3 6
50

Figure S6E

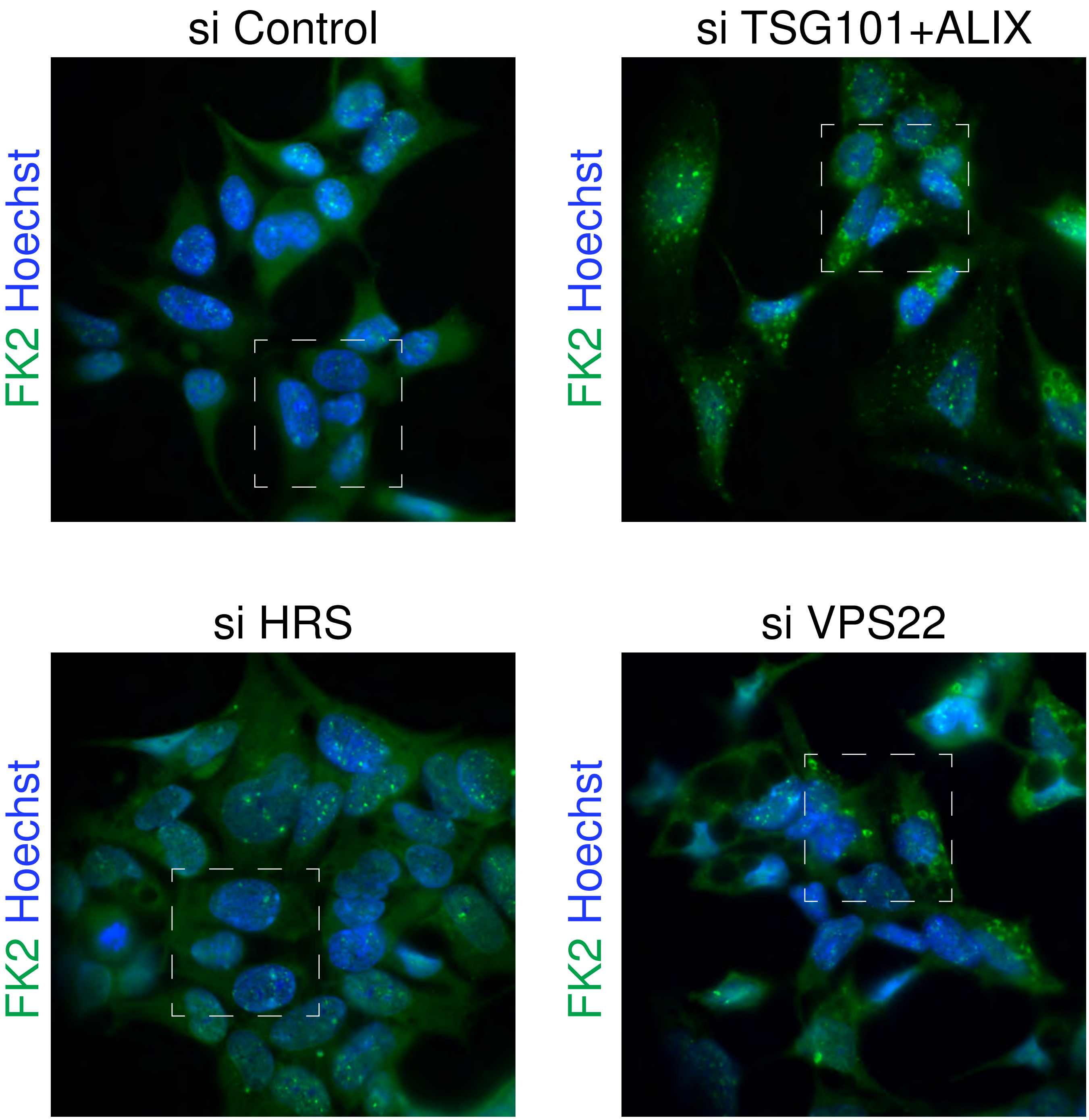

si Control
si TSG101+ALIX
si HRS
si VPS22
FK2 Hoechst
FK2 Hoechst
FK2 Hoechst
FK2 Hoechst

Figure S7

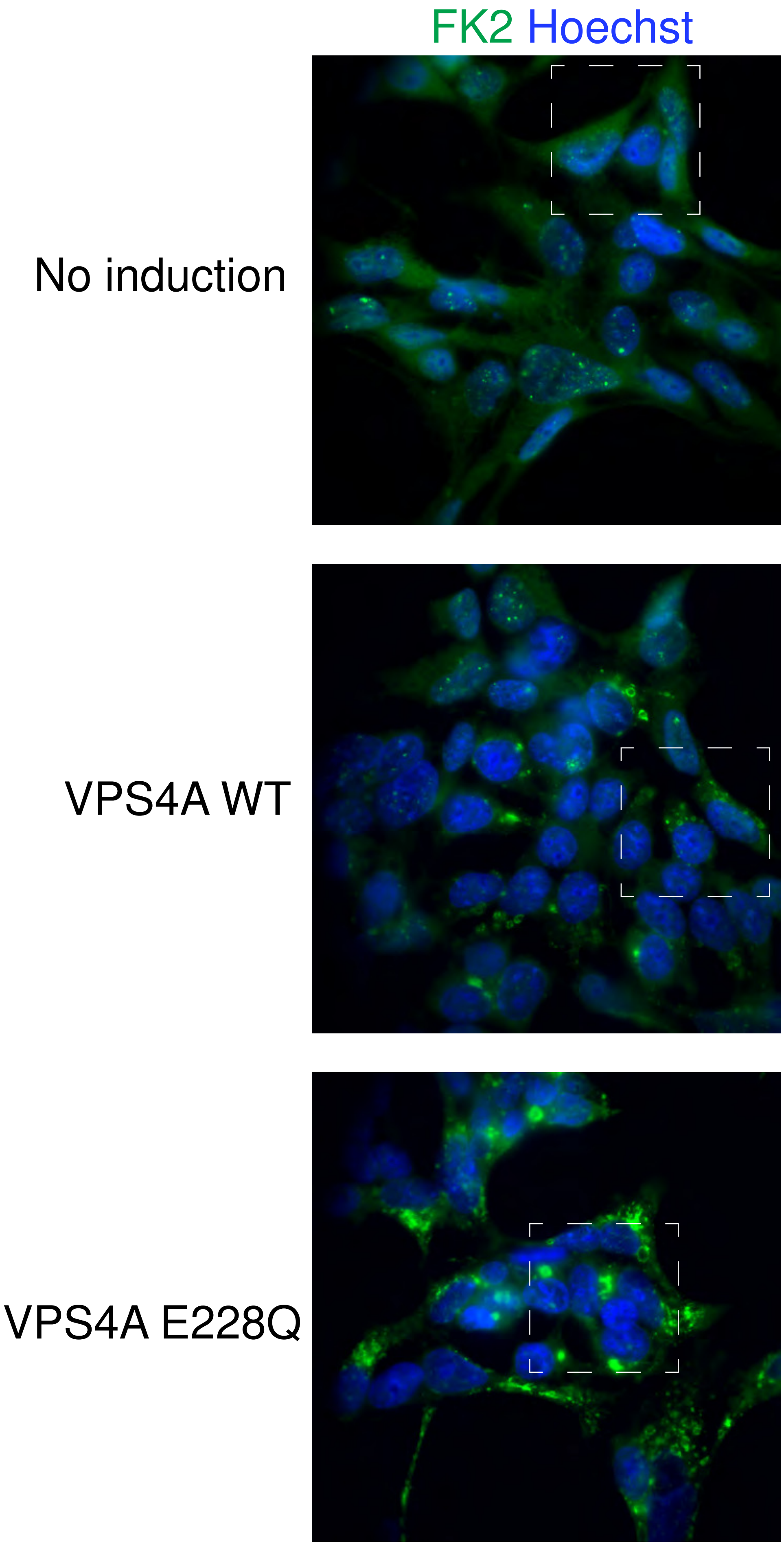

Supplement: S1 Raw Images — (PDF) [file pbio.3001361.s010.pdf]
