# Supplementary material for: A conserved ubiquitin- and ESCRT-dependent pathway internalizes human lysosomal membrane proteins for degradation
Source: PLoS Biol. 2021 Jul 23;19(7):e3001361. doi: 10.1371/journal.pbio.3001361 (PMC8337054; doi:10.1371/journal.pbio.3001361)
Supplement: S1 Table — (DOCX) [file pbio.3001361.s011.docx]

**S1 Table**

| **S1A Table: Mammalian cell lines used in this study** | | |
| --- | --- | --- |
| ***Cell lines*** | ***Description*** | ***reference/source*** |
| Human HEK293 | CRL-1573 | ATCC |
| Human HEK293T | CRL-3216 | ATCC |
| Human HeLa | CCL-2 | ATCC |
| Human HEK293, GFP-RNF152 | pHAGE2-EF1α-EGFP-RNF152-IRES-Puro | This study |
| Human HEK293, GFP-RNF152 (4C🡪S) | pHAGE2-EF1α-EGFP-RNF152 (4C🡪S)-IRES-Puro | This study |
| Human HEK293, GFP-RNF152 (8K🡪R) | pHAGE2-EF1α-EGFP-RNF152 (8K🡪R)-IRES-Puro | This study |
| Human HEK293, ATG5KO, GFP-RNF152 | ATG5 CRISPR-Cas9 knockout, pHAGE2-EF1α-EGFP-RNF152-IRES-Puro | This study |
| Human HEK293, ATG7KO, GFP-RNF152 | ATG7 CRISPR-Cas9 knockout, pHAGE2-EF1α-EGFP-RNF152-IRES-Puro | This study |
| Human HEK293, GFP-RNF152, FLAG-Lyso | pHAGE2-EF1α-EGFP-RNF152-IRES-mCherry, pLJC5-TMEM192-2XFLAG-Puro (Addgene 102929) | This study (Abu-Remaileh et al. 2017) |
| Human HEK293, GFP-RNF152, HA-Lyso | pHAGE2-EF1α-EGFP-RNF152-IRES-mCherry, pLJC5-TMEM192-3XHA-Puro (Addgene 102930) | This study (Abu-Remaileh et al. 2017) |
| Human HEK293, GFP-RNF152, HA-Vps4A | pHAGE2-EF1α-EGFP-RNF152-IRES-mCherry, pCW57.1-HA-Vps4A-Puro | This study |
| Human HEK293, GFP-RNF152, HA-Vps4A E228Q | pHAGE2-EF1α-EGFP-RNF152-IRES-mCherry, pCW57.1-HA-Vps4A (E228Q)-Puro | This study |
| Human HEK293, 3XFLAG-RNF152 | pHAGE2-EF1α-3XFLAG-RNF152-IRES-Puro | This study |
| Human HEK293, 3XFLAG-RNF152 (QK🡪AA) | pHAGE2-EF1α-3XFLAG-RNF152 (QK🡪AA)-IRES-Puro | This study |
| Human HEK293, LAPTM4A-GFP | pHAGE2-EF1α-LAPTM4A-EGFP-IRES-Puro | This study |
| Human HEK293, LAPTM4A (3PY)-GFP | pHAGE2-EF1α-LAPTM4A (3PY)-EGFP-IRES-Puro | This study |
| Human HEK293, LAPTM4A-GFP, HA-Vps4A | pHAGE2-EF1α-LAPTM4A-EGFP-IRES-mCherry, pCW57.1-HA-Vps4A (E228Q)-Puro | This study |
| Human HEK293, LAPTM4A-GFP, HA-Vps4A E228Q | pHAGE2-EF1α-LAPTM4A-EGFP-IRES-mCherry, pCW57.1-HA-Vps4A (E228Q)-Puro | This study |
| Human HEK293T, FLAG-Lyso | pLJC5-TMEM192-2XFLAG-Puro (Addgene 102929) | This study (Abu-Remaileh et al. 2017) |
| Human HEK293T, HA-Lyso | pLJC5-TMEM192-3XHA-Puro (Addgene 102930) | This study (Abu-Remaileh et al. 2017) |
| Human HeLa, GFP-RNF152 | pHAGE2-EF1α-EGFP-RNF152-IRES-Puro | This study |

| **S1B Table: Mammalian plasmids used in this study** | | | |
| --- | --- | --- | --- |
| ***Vector*** | ***Insert*** | ***description*** | ***reference/source*** |
| pEGFP-C1 | RNF152 | CMV promoter, N-terminal GFP | This study |
| pBICEP-CMV2-3XFLAG | RNF152 | CMV promoter, N-terminal 3XFLAG | This study |
| pcDNA3.1(-) | RNF152 | CMV promoter | This study |
| pHAGE2-IRES-Puro | EGFP | EF1α promoter, puromycin selection | This study |
| pHAGE2-IRES-Puro | EGFP-RNF152 | EF1α promoter, puromycin selection | This study |
| pHAGE2-IRES-Puro | LAPTM4A-EGFP | EF1α promoter, puromycin selection | This study |
| pHAGE2-IRES-Puro | LAPTM4A (3PY)-EGFP | EF1α promoter, puromycin selection | This study |
| pHAGE2-IRES-Puro | EGFP-RNF152 (4C🡪S) | EF1α promoter, puromycin selection | This study |
| pHAGE2-IRES-Puro | EGFP-RNF152  (8K🡪R) | EF1α promoter, puromycin selection | This study |
| pHAGE2-IRES-Puro | 3XFLAG-RNF152 | EF1α promoter, puromycin selection | This study |
| pHAGE2-IRES-Puro | 3XFLAG-RNF152 (QK🡪AA) | EF1α promoter, puromycin selection | This study |
| pHAGE2-IRES-mCherry | EGFP-RNF152 | EF1α promoter, mCherry selection | This study |
| pHAGE2-IRES-mCherry | LAPTM4A-EGFP | EF1α promoter, mCherry selection | This study |
| pEGFP-C1 | Vps4 E228Q | CMV promoter, N-terminal GFP | Votteler et al. 2016  Addgene 80351 |
| pCMV-HA | HA-Vps4A E228Q | CMV promoter, N-terminal HA | This study |
| pCW57.1 | HA-Vps4A WT | Tet-on promoter | This study |
| pCW57.1 | HA-Vps4A E228Q | Tet-on promoter | This study |
| pCMV-HA-Ub |  | CMV promoter, three repeats of HA-Ub | This study |
| pLJC5 | TMEM192-2XFLAG | UbC promoter | Abu-Remaileh et al. 2017  Addgene 102929 |
| pLJC5 | TMEM192-3XHA | UbC promoter | Abu-Remaileh et al. 2017  Addgene 102930 |
| pSpCas9(BB)-2A-Puro (PX459) |  | CRISPR-Cas9 knockout | Ran et al. 2013  Addgene, 48139 |
| psPAX2 |  | Lentiviral packaging plasmid | Addgene 12260 |
| pMD2.G |  | VSV-G envelope | Addgene 12259 |
| pLKO.1 |  | shRNA knockdown | Addgene 8453 |
| ***Overexpression plasmids used in the cycloheximide chase screen*** | | | |
| pcDNA3-EGFP | CLCN7 | CMV promoter, C-terminal GFP | This study. CDS from Origene. |
| pCMV6-AC-GFP | OSTM1 | CMV promoter, C-terminal turboGFP | This study, Origene |
| pCMV-SPORT6 | p40-EGFP | CMV promoter, C-terminal GFP | Gift from Dr. M Boonen. (Boonen et al. 2006) |
| pEGFP-C1 | hPAT1 (SLC36A1) | CMV promoter, N-terminal GFP | This study, CDS from Origene |
| pEGFP-C1 | TMEM192 | CMV promoter, N-terminal GFP | Gift from B. Schröder. (Schröder et al. 2010) |
| pEGFP-C2 | TRPML1 | CMV promoter, N-terminal GFP | Dong et al. 2008. |
| pEGFP-N1 | CTNS | CMV promoter, C-terminal GFP | This study, CDS from DNASU. |
| pEGFP-N1 | hSpinster1 | CMV promoter, C-terminal GFP | This study. (Rong et al. 2011) |
| pEGFP-N1 | SCARB2 | CMV promoter, C-terminal GFP | This study, CDS from DNASU. |
| pReceiver-M03-EGFP | PQLC2 | CMV promoter, C-terminal GFP | This study  GeneCopoeia |
| pReceiver-M03-EGFP | TTYH2 | CMV promoter, C-terminal GFP | This study  GeneCopoeia |
| pReceiver-M03-EGFP | TTYH3 | CMV promoter, C-terminal GFP | This study  GeneCopoeia |
| pReceiver-M29-EGFP | C9orf91 | CMV promoter, N-terminal GFP | This study  GeneCopoeia |
| pReceiver-M29-EGFP | ITM2C | CMV promoter, N-terminal GFP | This study  GeneCopoeia |
| pReceiver-M29-EGFP | MFSD8 | CMV promoter, N-terminal GFP | This study  GeneCopoeia |
| pReceiver-M29-EGFP | OCA2 | CMV promoter, N-terminal GFP | This study  GeneCopoeia |
| pReceiver-M29-EGFP | SLC38A7 | CMV promoter, N-terminal GFP | This study  GeneCopoeia |
| pReceiver-M29-EGFP | STARD3 | CMV promoter, N-terminal GFP | This study  GeneCopoeia |
| pReceiver-M29-EGFP | TMEM106B | CMV promoter, N-terminal GFP | This study  GeneCopoeia |
| pReceiver-M29-EGFP | TMEM127 | CMV promoter, N-terminal GFP | This study  GeneCopoeia |
| pReceiver-M29-EGFP | TMEM175 | CMV promoter, N-terminal GFP | This study  GeneCopoeia |
| pReceiver-M55-mCherry | LAPTM5 | CMV promoter, N-terminal mCherry | This study  GeneCopoeia |
| pReceiver-M55-mCherry | TMEM55B | CMV promoter, N-terminal mCherry | This study  GeneCopoeia |
| pReceiver-M56-mCherry | LAPTM4A | CMV promoter, C-terminal mCherry | This study  GeneCopoeia |
| pReceiver-M56-mCherry | SLC7A14 | CMV promoter, C-terminal mCherry | This study  GeneCopoeia |

| **S1C Table: Yeast strains and Plasmids used in this study** | | | |
| --- | --- | --- | --- |
| ***S. cerevisiae strains*** | | | |
| ***strain*** | ***name*** | ***genotype*** | ***reference/source*** |
| SEY6210 | wild type | Matα*, leu1-3, 112 ura3-52 his3-200, trp1-901 lys2-801 suc2-D9* | Robinson et al., 1988 |
| SEY6210.1 | wild type | Matα*, leu1-3, 112 ura3-52 his3-200, trp1-901 lys2-801 suc2-D9* | Robinson et al., 1988 |
| YXY813 | *pep4*Δ | 6210.1, *pep4*Δ::KAN | This study |
| YML377 | *vps27*Δ | 6210, *vps27*Δ::HIS3 | Li et al., 2015 |
| YML068 | *vps4*Δ | 6210.1, *vps4*Δ::TRP1 | Li et al., 2015 |
| YXY624 | *vps23*Δ | 6210.1, *vps23*Δ::TRP1 | This study |
| YXY1031 | *vps22*Δ | 6210, *vps22*Δ::KAN | This study |
| YXY625 | *snf7*Δ | 6210.1, *snf7*Δ::TRP1 | This study |
| YXY1030 | *bro1*Δ | 6210, *bro1*Δ::KAN | This study |
| ***S. cerevisiae expression plasmids*** | | | |
| ***vector*** | ***Insert*** | ***description*** | ***reference/source*** |
| pRS415 | GFP-RNF152 | ADH1 promoter, N-terminal GFP | This study |

**References:**

Abu-Remaileh M, Wyant GA, Kim C, et al. Lysosomal metabolomics reveals V-ATPase- and mTOR-dependent regulation of amino acid efflux from lysosomes. *Science*. 2017;358(6364):807-813. doi:10.1126/science.aan6298

Dong XP, Cheng X, Mills E, et al. The type IV mucolipidosis-associated protein TRPML1 is an endolysosomal iron release channel. *Nature*. 2008;455(7215):992-996. doi:10.1038/nature07311

Li M, Rong Y, Chuang YS, Peng D, Emr SD. Ubiquitin-dependent lysosomal membrane protein sorting and degradation. *Mol Cell*. 2015;57(3):467-478. doi:10.1016/j.molcel.2014.12.012

Ran FA, Hsu PD, Wright J, Agarwala V, Scott DA, Zhang F. Genome engineering using the CRISPR-Cas9 system. *Nat Protoc*. 2013;8(11):2281-2308. doi:10.1038/nprot.2013.143

Robinson JS, Klionsky DJ, Banta LM, Emr SD. Protein sorting in Saccharomyces cerevisiae: isolation of mutants defective in the delivery and processing of multiple vacuolar hydrolases. *Mol Cell Biol*. 1988;8(11):4936-4948. doi:10.1128/mcb.8.11.4936

Rong Y, McPhee CK, Deng S, et al. Spinster is required for autophagic lysosome reformation and mTOR reactivation following starvation [published correction appears in Proc Natl Acad Sci U S A. 2011 Jul 5;108(27):11297. McPhee, Christina [corrected to McPhee, Christina K]; Baehreck, Eric H [corrected to Baehrecke, Eric H]]. *Proc Natl Acad Sci U S A*. 2011;108(19):7826-7831. doi:10.1073/pnas.1013800108

Schröder B, Wrocklage C, Hasilik A, Saftig P. Molecular characterisation of 'transmembrane protein 192' (TMEM192), a novel protein of the lysosomal membrane. *Biol Chem*. 2010;391(6):695-704. doi:10.1515/BC.2010.062

Votteler J, Ogohara C, Yi S, et al. Designed proteins induce the formation of nanocage-containing extracellular vesicles. *Nature*. 2016;540(7632):292-295. doi:10.1038/nature20607
