# Supplementary material for: A conserved ubiquitin- and ESCRT-dependent pathway internalizes human lysosomal membrane proteins for degradation
Source: PLoS Biol. 2021 Jul 23;19(7):e3001361. doi: 10.1371/journal.pbio.3001361 (PMC8337054; doi:10.1371/journal.pbio.3001361)
Supplement: Attachment — Submitted filename: Reviewers commons rebuttal letter.pdf [file pbio.3001361.s012.pdf]

Reviewer #1:

1. As a specific (but not the only) example of over-reaching in generalizing the findings, the abstract ends with the following statement: "Thus, our study uncovered a conserved mechanism to down-regulate lysosome membrane proteins." My concern is that although this mechanism might be generalizable, the authors have only presented data for RNF152.

Thank you for the comment. We now included a second lysosome membrane protein, LAPTM4A (**figure 6-7**). We demonstrated that both endogenous LAPTM4A and GFP-tagged LAPTM4A are quickly degraded. With lyso-IP, we confirmed that endogenous LAPTM4A is enriched in the lysosome fraction. Further analysis indicated that LAPTM4A contains 3 PY motifs (PPxY or LPxY) at its C-terminus. The PY motif is responsible for recruiting a cytosolic E3 ligase NEDD4-1 to the membrane and ubiquitinating LAPTM4A. Either mutating the PY motif or knockdown NEDD4-1 stabilized LAPTM4A (**figure 6**). Lastly, we showed that knocking down CHMP4A+CHMP4B and overexpression of a dominant-negative VPS4A mutant can stabilize ubiquitinated LAPTM4A (**figure 7**).

Together with RNF152, our analysis of LAPTM4A provided another line of evidence to support that the ubiquitin- and ESCRT-dependent degradation of lysosome membrane proteins is a general mechanism.

2. There is a complete reliance on over-expressed, GFP-tagged RNF152. There is no demonstration that the endogenously expressed protein undergoes such high rates of turnover. It is thus possible that the data does not reflect the normal turnover pathway for this protein.

We agree. To address the issue, we generated an antibody against RNF152 (**supporting figure 1**). Due to its fast degradation and probably the low sensitivity of the antibody, the endogenous RNF152 is too low to be detected. However, we could detect the endogenous RNF152 after using BafA1 to stop RNF152 degradation. To rule out the possibility that the GFP tag is causing RNF152 degradation, we tested the non-tagged RNF152 at two expression levels, including a low-level leaky expression from a TET-ON promoter (i.e., without the addition of doxycycline) and overexpression from a CMV promoter. Under both conditions, RNF152 degraded.

Moreover, we purchased a LAPTM4A antibody and used it to demonstrate that endogenous LAPTM4A is lysosome-localized and quickly degraded (**figure 6B-C**). Taken together, we have collected strong evidence to support that both RNF152 and LAPTM4A are turned over quickly under normal conditions.

3. In Figure 2B, why is the loss of full-length RNF152-GFP not accompanied by an increase in the signal for free GFP during these pulse-chase experiments?

Indeed, compared to yeast vacuole, GFP is less stable inside human lysosomes and can be partially degraded by lumenal proteases. This is the reason for not observing GFP accumulation in **Figure 2B**. Two possible reasons may contribute to the GFP degradation and no accumulation: 1) Human cell lines were grown at 37 °C, whereas yeast cells were grown at 30 °C. At 37 °C, GFP is less resistant to the lumenal proteases. This is true even in yeast. 2) Human lysosome is more acidic than the yeast vacuole (pH~4.5 vs. pH 5.5). Consequently, GFP fluorescence is quenched inside the human lysosome, but still fluorescent in yeast vacuole. The lower pH may also contribute to the degradation of GFP.

4. Figure 2E: Were all of the pairs of Input and IP immunoblots subject to the same exposure and image adjustments?

Yes, they were on the same membrane and subjected to the same adjustment. We included all the original data as **supporting figure 2**.

5. *Figure 3C-E: The RNF152 mutants have slowed but not eliminated degradation. Is this dependent on their association with or ubiquitination by the endogenously expressed RNF152?*

This is a great question. For the 8KR mutant, the possible reason for not having a complete block is because GFP might contribute additional lysines for ubiquitination. As to the 4CS mutant, we agree with this reviewer that endogenous RNF152 could ubiquitinate the 4CS mutant. In fact, we recently performed a whole-genome CRISPR knockout screening to identify genes important for the degradation of GFP-RNF152 (**supporting figure 3**). Our #1 hit was RNF152, which is consistent with the hypothesis that endogenous RNF152 can ubiquitinate GFP-RNF152, including the 4CS mutant. Other hits include many vATPase components, which also makes sense because lumenal pH is critical for the lysosome protease activity.

Due to COVID19, we have not finished our analysis of the screen results. And it is beyond the scope of this manuscript to include the screen. We will complete the study and report the screen in a separate manuscript later.

6. Methods section indicates that t-tests were performed for all statistics. However, many experiments contain multiple comparisons and are thus ideally suited to t-tests. The authors should either justify the use of t-tests or provide a more suitable statistical analysis.

Thank you for the comments. Per this reviewer's request, we have performed the one-way ANOVA analysis for all multiple comparisons, and the figures have been updated.

7. *Although the model in Figure 7 shows the E3 (RNF152) ubiquitinating other proteins and promoting their ESCRT-dependent sorting into ILVs, this study did not identifying any such clients of RNF152.*

Indeed, we only demonstrated that the auto-ubiquitination RNF152 leads to fast degradation of the protein in our initial submission. In this revision, we included LAPTM4A, which can be ubiquitinated by a cytosolic E3 ligase NEDD4-1. LAPTM4A has 3 PY motifs that can recruit NEDD4-1 to the lysosome membrane(**figure 6**). In figure 7D (now **8D** in the revision), we proposed a model that lysosomes in both yeast and humans contain E3 ligase systems to ubiquitinate and down-regulate their membrane proteins. For yeast, we have identified three independent vacuolar E3 ligase systems in our previous studies (Li et al., 2015a; Li et al., 2015b; Yang et al., 2018; Yang et al., 2020). In this study, we identified RNF152 and NEDD4-1 that function on the human lysosome.

With the addition of NEDD4-1 and its substrate LAPTM4A, we significantly strengthened the evidence to support the model in **figure 8D.**

Minor

Page 3: "Without treatment, almost all types of LSD patients will develop severe neurodegeneration in the central nervous system." This statement is misleading as there are multiple forms of LSDs that do not result in neurodegeneration and it is only these LSDs which

can be successfully treated via enzyme replacement therapies. Unfortunately, the neuropathic LSDs remain largely untreatable due largely to issues of blood brain barrier permeability.

We rephrased the sentence to "Without treatment, many LSD patients will develop severe neurodegeneration symptoms."

Page 3: "As we age, the lysosome membrane gradually accumulates damaged proteins and loses its activity, which dampens the cell's ability to remove pathogenic protein aggregates and damaged organelles, eventually leading to cell death and inflammation (Carmona-Gutierrez et al., 2016; Cheon et al., 2019; Yambire et al., 2019)." The references provided do not provide sufficient direct support for this broad statement.

We added another recent review on this topic to justify the statement (Nixon, 2020). Here is a direct quote from the Nixon, 2020:

"Lysosomal membrane permeabilization (LMP) is a possible outcome of cumulative insults of lysosomal aging … These routes include damage from free radicals, membrane incorporation of damaged proteins and oxidized lipids, and ionic shifts that alter osmotic equilibrium, and increased lysosomal volume that is associated with enhanced fragility and LMP . "

As mentioned in these references, damaged lysosomes lead to autophagy defect, apoptosis and inflammation.

Page 10: STED imaging results (currently "data not shown") should be supported by showing the relevant data.
Please see **supporting figure 5** for the STED imaging results.

Camera and objective information should be provided for microscopy studies.

We added the information in the **Microscopy and image processing**" section of the **Materials and Methods**.

Reviewer #2:

1. The authors have found that among 30 LMPs, three LMPs, LAPTM4A, RNF152, and OCA2, have half-lives less than 9 hours. RNF152 is a ubiquitin ligase and the authors showed that auto-ubiquitination is important for the recognition by the ESCRT machinery. Can the authors speculate how the ligase activity of RNF152 is regulated? Also, is similar mechanism involved in LAPTM4A and OCA2 turnover? Are these two proteins also ubiquitinated?

The regulation of RNF152 E3 ligase activity is a fascinating question, and not much is known. RNF152 has been demonstrated to be a negative regulator of the mTOR signaling pathway by ubiquitinating RagA and Rheb GTPases and thus inhibiting their abilities to activate mTORC1(Deng et al., 2019; Deng et al., 2015). Physiologically speaking, it makes sense to keep RNF152 at a low level through autoubiquitination and the ESCRT-dependent degradation under growing conditions. We have confirmed that RNF152 does interact with RagA through immunoprecipitation(IP) experiments (**supporting figure 4**). Importantly, we also observed that the RNF152-interacting RagA is indeed poly-ubiquitinated. Because the role of RNF152 in the mTOR signaling pathway has been published, we did not pursue this question further.

In this revision, we demonstrated that LAPTM4A is also down-regulated at a ubiquitin- and ESCRT-dependent manner (**figures 6-7**). Please see our responses to reviewer #1, major points 1&2, for more details. We did not pursue OCA2 because it is a melanosome-specific protein and does not exist on lysosomes.

2. The authors should at least demonstrate that endogenous RNF152 levels and turnover are also regulated by ESCRT III and VPS4, using the stable cell lines the authors have already made. All of the mammalian cell experiments are performed using overexpression of RNF152, and an endogenous experiment would inspire confidence that the author's findings are not an artifact of over-expression.

As stated in our response to reviewer #1, major point 2, the protein level of endogenous RNF152 is too low to be detected by our RNF152 antibody (**supporting figure 1**). However, we included LAPTM4A in this revision. As shown in **figure 7G-H**, the endogenous LAPTM4A is stabilized after knocking down CHM4A+4B.

3. While the authors showed that the K->R and C->S mutants of RNF152 have increased stability, it would be more compelling if they could perform an IP using HA-ubiquitin to prove this effect is due to a loss/reduction of RNF152 ubiquitination and not due to other changes in the protein. Another concern is whether mutating 8 lysine or 4 cysteine residues simultaneously would affect the folding of the protein, leading to abnormal aggregation in the cell.

Per this reviewer's request, we performed the IP and ubiquitin blot experiments. As shown in **figure 3F** of this revision, 4CS and 8KR mutants have significantly less poly-ubiquitination. Furthermore, we did not observe protein aggregation for the 8KR and 4CS mutants on the gel.

4. For some of the data, statistical analysis is missing:
a. All of the cycloheximide chase experiments.

We added all the statistical analyses in this revision.

b. statistical significance for the puncta vs membrane GFP signal data shown in figure 6f

We added the statistical analysis for **figure 6F**.

c. The flow cytometry data

For flow cytometry experiments in **Figures 2C, 4E, 5C**, and **5H**, we collected at least 10,000 events for each sample group and presented the distribution of their intensity. Statistical analysis is uncommon for this type of data.

5. Fig. 4A and Fig. S2A, why MG132 treatment affects the levels of free GFP if it's inside of the lysosome?

This is a great question. In addition to inhibiting the proteasome function, MG132 is known to mildly affect some lysosomal cathepsins (Kisselev and Goldberg, 2001; Rock et al., 1994; Shirley et al., 2005). As a result, free GFP becomes more stable inside the human lysosome.

6. Make sure that the figures are properly referenced in the text, there is one instance where the authors referenced figure 2d, when they clearly meant to reference figure 2e, and figure 2e where the authors meant to reference figure 2f etc.

We fixed them. Thank you for catching all these issues.

Minor Comments:

1. In figure 1A, at CHX 3h, there's ~40% reduction of GFP-RNF152, however, in the rest of the figures, such as figure 2B， at CHX 2h, there's ~70-80% reduction of GFP-RNF152. How to explain the difference in the kinetics?

In **Figure 1A**, GFP-RNF152 was transiently expressed from a pEGFP-C1 plasmid. We used stable cell lines for the rest of the paper. There was a considerable variation of GFP-RNF152 expression levels with the transient expression plasmid, and some cells were exceedingly bright. It is possible that when cells are overwhelmed with overexpression, the degradation machinery could be saturated, and the degradation is slower.

2. In figure 2F, it is hard to differentiate when the underline for input ends and the underline for IP begins unless the reader zooms in, please separate them a bit more.

We fixed the issue. Thank you for pointing this out.

3. Fig. 4F, it's very hard to see the red and green signals, maybe get rid of the DAPI channel increase the intensity for both green and red channels, and zoom in?

We did zoom in for **figure 4F** and enhanced the intensity for red and green channels. Thank you!

4. Scale bars are missing in the insert images in figure 1C, figure 4G and figure 6E.

We fixed the problem. Thank you!

5. In figure S1, the labels do not match with the blot for GFP-TMEM106B time points.

We fixed the problem. Thank you!

Reviewer #3:

Major comments:

1. The writing of the manuscript including the abstract could be further polished. The manuscript in its present form appears to be a technical report that does not sufficiently convey the significance of this study.

Thank you for providing your honest opinion. We agree that our writing could be better. We revised the manuscript several rounds. We also ask several colleagues in our department who are native speakers to edit the manuscript. It is now more concise and easier to read.

2. Cyclohexamide is commonly used in studying the half-lives of proteins of interests. This is not a new method authors developed in the first place.

We have changed to wording from "developed" to "performed" in the abstract.

3. The data of protein turnover was presented by plotting the relative level of proteins as a function of time. But the use of degradation kinetics was all over the place in the manuscript, which is inappropriate scientifically. The authors should first generate fit to first-order decay to acquire a degradation rate constant, k (min-1) and calculate half-life (T1/2) from there.

We have fit our quantifications to first-order decay to calculate the protein half-life per this reviewer's request. All the half-lives are now included in the revised figures.

4. What are the functional consequences of RNF152 degradation? What are the biological impacts at both lysosomal and cellular levels in RNF152-depleted cells?

RNF152 is a negative regulator of the mTOR signaling pathway by ubiquitinating RagA and Rheb GTPases and thus inhibiting their abilities to activate mTORC1(Deng et al., 2019; Deng et al., 2015). Physiologically speaking, keeping RNF152 at a low level helps to maintain the mTORC1 in an active state. We have confirmed that RNF152 does interact with RagA through IP experiments (**supporting figure 4**). Importantly, we observed that the RNF152-interacting RagA is indeed poly-ubiquitinated. Because the role of RNF152 in the mTOR signaling pathway has been published, we did not pursue this question further.

5. Given the rapid turnover of RNF152 at basal state, one can predict that this protein may become functionally important under specific circumstances, for example, certain stress. This aspect is worth exploring.

We agree. As we stated above, RNF152 is a negative regulator of the mTOR signaling pathway, which has been well-characterized by others recently (Deng et al., 2019; Deng et al., 2015). Because the roles of RNF152 have been reported and the focus of this paper is to study how LMPs are down-regulated, we did not further investigate the functional importance of RNF152 under stress conditions.

6. The authors chose RNF152 over OCA2, a melanosome-specific protein. However, OCA2 was shown to colocalize with LAMP2 much better than RNF152.

As stated in our manuscript and by this reviewer, OCA2 is a melanosome-specific protein. Melanosome is a lysosome-like organelle, but not the lysosome. Instead of OCA2, we characterized LAPTM4A in detail and included the data in this revision (**Figure 6-7**).

Minor comments:

1. Mislabeling and typo errors detected in the text:
a. Page 7 "As expected, the full-length GFP-RNF152 and other lysosomal proteins such as LAMP2 and cathepsin D (CTSD) were enriched by Lyso-IP. In contrast, PDI (ER), Golgin160 (Golgi), EEA1 (endosomes), and GAPDH (cytosol) were not enriched (Figure 2D)." - should be Figure 2E instead.

b. Page 7 "Our result confirmed that the lysosome population of GFP-RNF152 is quickly turned over, while LAMP2 is very stable on the lysosome (Figure 2E)." - should be Figure 2F instead.

c. Page 14 "knocking down either TSG101 or both TSG101 and RNF152 only had a minor impact on the degradation kinetics of GFP-RNF152 (Figure S3A-B)." - should be ALIX instead of RNF152.

We fixed all these typos. Thank you!

2. Stable cells expressing GFP-RNF152 or 3xFLAG-RNF152 were primarily used in this study. It will be useful to perform some experiments by examining the endogenous counterpart using antibodies against RNF152. For example, Figure 2D and 2E.

We raised an antibody against RNF152. However, the endogenous RNF152 level is too low to be detected. The only condition to detect the endogenous RNF152 was treating cells with BafA1 to stop the RNF152 degradation (**supporting figure 1**). In addition, we also characterized the non-tagged RNF152 and showed it was also quickly degraded (**supporting figure 1 and new figure 2B**). Please also see our response to reviewer #1, major point 2, for more information.

Besides RNF152, we also showed that endogenous LAPTM4A is quickly degraded (**Figure 6B**).

3. For all the flow cytometry analysis, the value of GFP intensity in respective graphs should be indicated.

We added the values. Thank you!

4. Statistics analysis was not performed on Figure 5D.

We performed the analysis as requested. It is **figure S3D** in this revision.

5. In Figure 6D and J, what are the reasons for the appearance of multiple peaks, particularly, by the red line?

We are not sure about the reason. It is possible that CHMP4A-4B knockdown efficiency (**figure 5C**) and the expression level of VPS4A (**figure 5H**) are different from cell to cell.

6. In Figure 3A, the question marks should be removed to avoid confusion. "Predicted" can be used instead if there is no direct evidence from mass spec analysis.

We replaced the question marks with “putative.” Thank you!

7. In Figure 3C, the authors identified two mutants including KR and CS that are refractory to degradation. It will be more insightful by showing the ubiquitination of these two mutants as in Figure 3B.

Thank you for the suggestion. We performed the ubiquitin blot and included the data in the revision (**figure 3F**)

References:

Deng, L., Chen, L., Zhao, L., Xu, Y., Peng, X., Wang, X., Ding, L., Jin, J., Teng, H., Wang, Y., *et al.* (2019). Ubiquitination of Rheb governs growth factor-induced mTORC1 activation. Cell Res *29*, 136-150.

Deng, L., Jiang, C., Chen, L., Jin, J., Wei, J., Zhao, L., Chen, M., Pan, W., Xu, Y., Chu, H., *et al.* (2015). The Ubiquitination of RagA GTPase by RNF152 Negatively Regulates mTORC1 Activation. Mol Cell.

Kisselev, A.F., and Goldberg, A.L. (2001). Proteasome inhibitors: from research tools to drug candidates. Chem Biol *8*, 739-758.

Li, M., Koshi, T., and Emr, S.D. (2015a). Membrane-anchored ubiquitin ligase complex is required for the turnover of lysosomal membrane proteins. J Cell Biol *211*, 639-652.

Li, M., Rong, Y., Chuang, Y.S., Peng, D., and Emr, S.D. (2015b). Ubiquitin-dependent lysosomal membrane protein sorting and degradation. Mol Cell *57*, 467-478.

Nixon, R.A. (2020). The aging lysosome: An essential catalyst for late-onset neurodegenerative diseases. Biochim Biophys Acta Proteins Proteom *1868*, 140443.

Rock, K.L., Gramm, C., Rothstein, L., Clark, K., Stein, R., Dick, L., Hwang, D., and Goldberg, A.L. (1994). Inhibitors of the proteasome block the degradation of most cell proteins and the generation of peptides presented on MHC class I molecules. Cell *78*, 761-771.

Shirley, R.B., Kaddour-Djebbar, I., Patel, D.M., Lakshmikanthan, V., Lewis, R.W., and Kumar, M.V. (2005). Combination of proteasomal inhibitors lactacystin and MG132 induced synergistic apoptosis in prostate cancer cells. Neoplasia *7*, 1104-1111.

Yang, X., Arines, F.M., Zhang, W., and Li, M. (2018). Sorting of a multi-subunit ubiquitin ligase complex in the endolysosome system. Elife *7*.

Yang, X., Zhang, W., Wen, X., Bulinski, P.J., Chomchai, D.A., Arines, F.M., Liu, Y.Y., Sprenger, S., Teis, D., Klionsky, D.J., *et al.* (2020). TORC1 regulates vacuole membrane composition through ubiquitin- and ESCRT-dependent microautophagy. J Cell Biol *219*.

Supporting figure 1

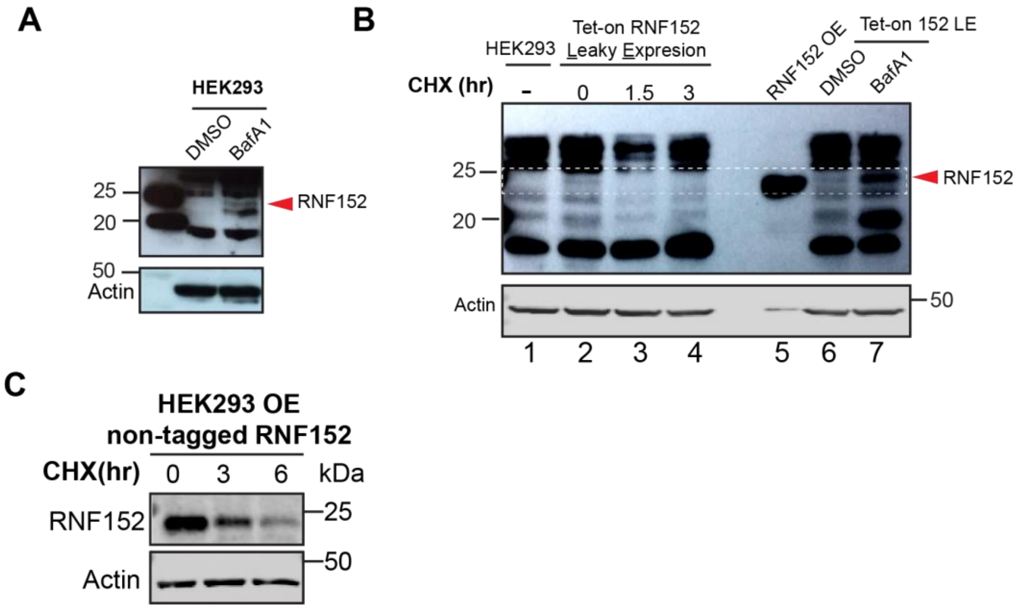

Non-tagged RNF152 is also quickly degraded.

(A) Endogenous RNF152 (arrow head) can only be detected after BafA1 treatment. (B) Lanes 2-4: CHX chase assay of RNF152 expressed from a leaky TET-ON promoter. Lane 5: RNF152 after Doxycycline induction, only 10% lysate loaded, Lanes 6-7: BafA1 treatment stabilized RNF152 expressed from the leaky TET-ON promoter. White box highlights the postion of RNF152. (C) CHX chase assay of transiently overexpressed RNF152 in HEK293 cells.

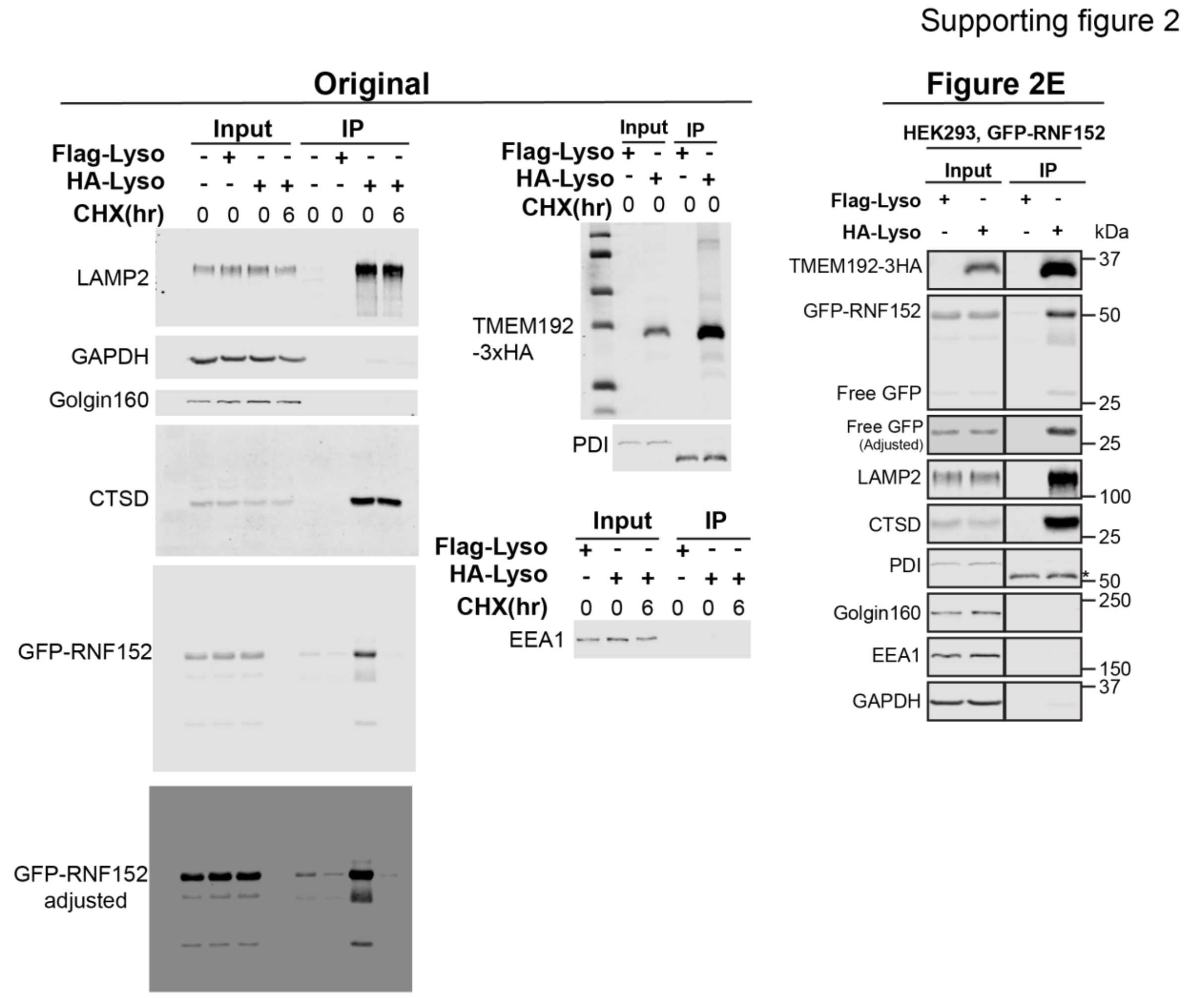

Original data of Figure 2E. All samples were from the same Lyso-IP experiments. They have been analyzed by several gels to be probed with 8 different antibodies. The intial experiments included a 6-hour CHX chase sample that was cropped out in figure 2E.

Supporting figure 3

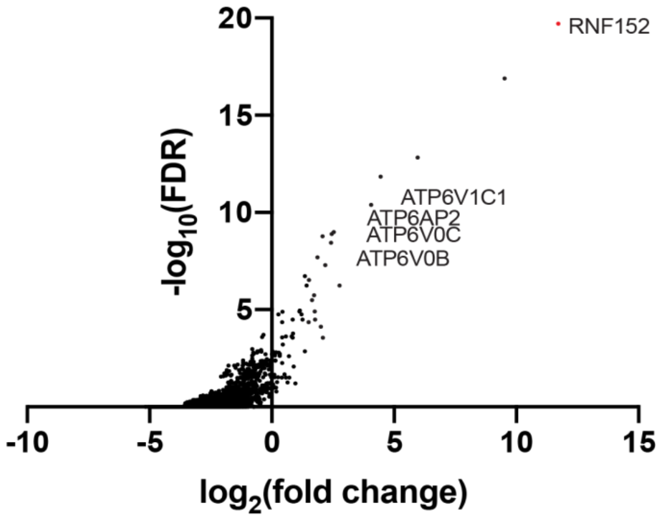

Hits from a whole-genome CRISPR knockout screen to identify genes important for the degradation of GFP-RNF152. RNF152 is the highest hit, other hits include vATPase components.

Supporting figure 4

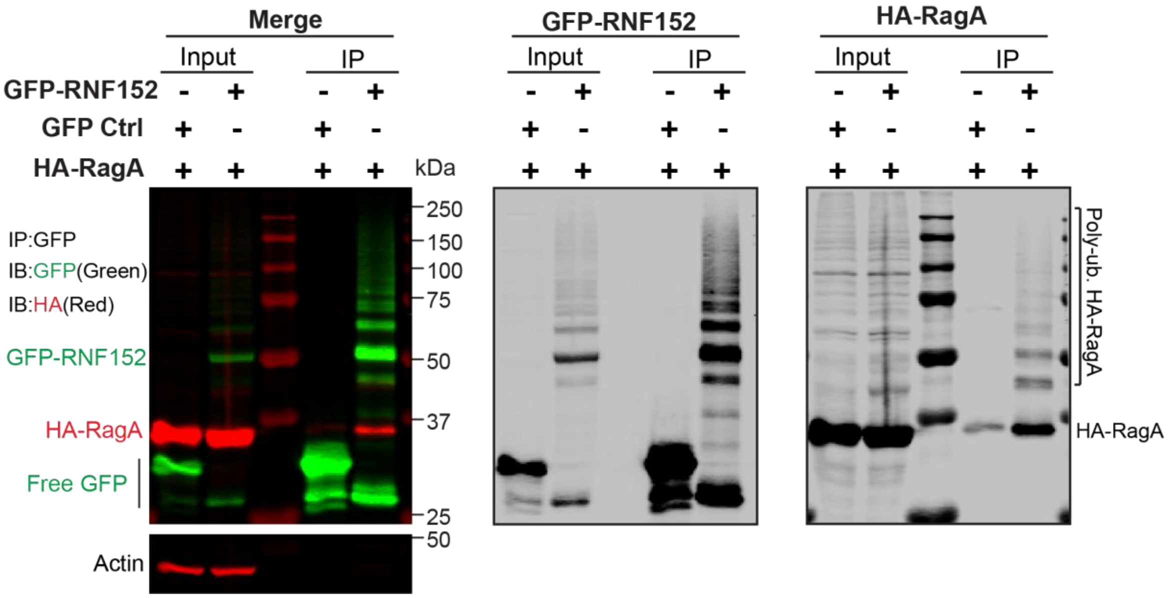

Co-IP experiment showing that GFP-RNF152 interacts with poly-ubiquitinated RagA

Supporting figure 5

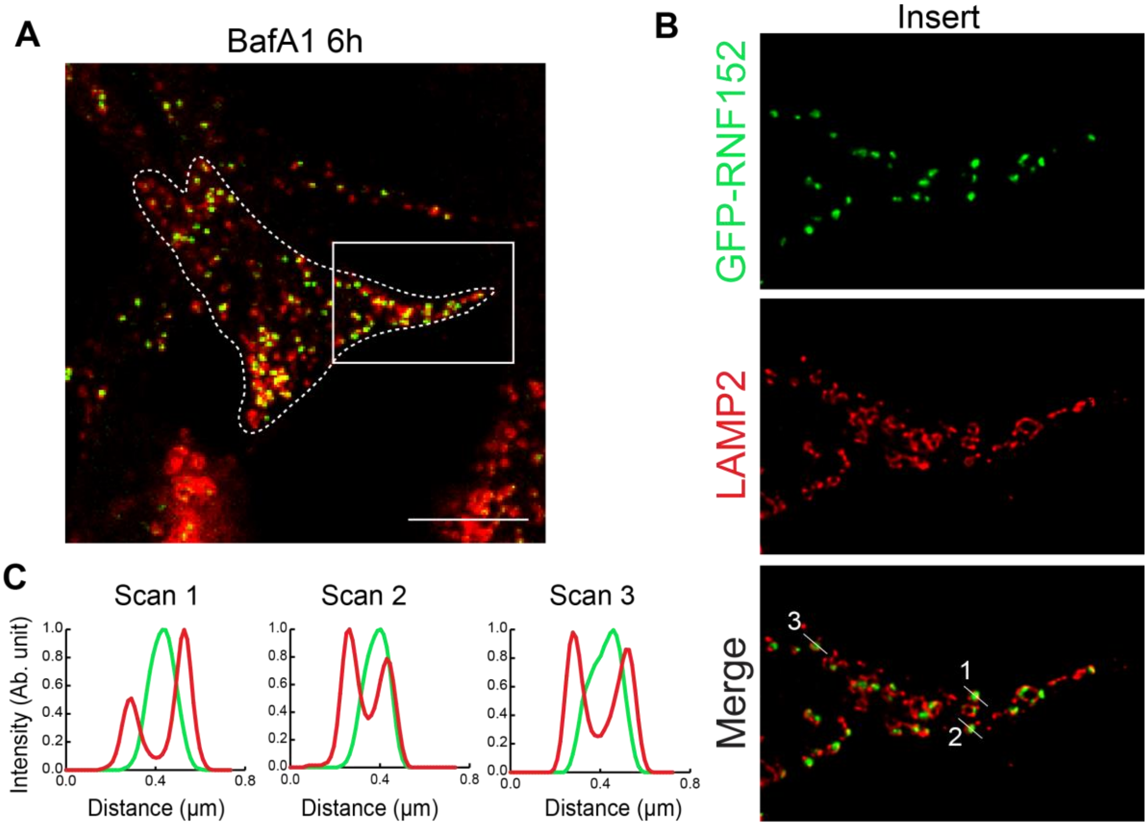

Images from STED super-resolution microscope to show GFP-RNF152 signal was localized inside the lysosome lumen after BafA1 treatment.

(A) Immunofluorescence of LAMP2 with GFP-RNF152 in HEK293 cells after BafA1 treatment. Scale bar=10 µm. (B) Insert of A. (C) Line scanning analysis was performed on three lysosomal structures in B.
