# Supplementary material for: A conserved ubiquitin- and ESCRT-dependent pathway internalizes human lysosomal membrane proteins for degradation
Source: PLoS Biol. 2021 Jul 23;19(7):e3001361. doi: 10.1371/journal.pbio.3001361 (PMC8337054; doi:10.1371/journal.pbio.3001361)
Supplement: Attachment — Submitted filename: Resonse to Reviewer #3.docx [file pbio.3001361.s014.docx]

Response to Rev. 3’s comments:

*1. Data presented in the response to reviewers should be included in the manuscript. If the reviewers need this information to assess the results, then other readers will appreciate them as well. That said, the preliminary screening data need not be added.*

Per this reviewer’s request, we generated two more supplemental figures (S2 and S3). With the exception of our preliminary screen, all the supporting data are now included.

*2. It remains unclear whether the RNF152 degradation observed with the over-expressed protein reflects the normal degradation pathway for the endogenous protein. Either way, it could be a useful tool. However, the authors should be more up front with this limitation if they cannot show that the endogenous protein is degraded in a similar manner.*

We agree. On the last paragraph of page 5, we included the description that endogenous RNF152 cannot be detected by our homemade antibody. “However, this antibody failed to detect the endogenous RNF152.”

*3. The authors state: "Without treatment,many LSD patients will develop severe neurodegeneration symptoms (Marques and Saftig, 2019)." This sentence in the introduction is problematic as it implies that there are treatments for the neurological aspects of lysosome storage diseases. Unfortunately, treatments such as enzyme replacement therapy are only effective for the non-neuronal symptoms.*

We deleted the “Without treatment”. It is true that many LSD patients will develop neurodegeneration phenotypes as they grow older.

*4. Page 27: Gene transfer with replication incompetent lentivirus is referred to as transduction rather than infection.*

We replaced the word “infection” with “transduction.” Thanks!
